# Coordinated Regulation of Membrane Homeostasis and Drug Accumulation by Novel Kinase STK-17 in Response to Antifungal Azole Treatment

**DOI:** 10.1128/spectrum.00127-22

**Published:** 2022-02-23

**Authors:** Chengcheng Hu, Mi Zhou, Xianhe Cao, Wei Xue, Zhenying Zhang, Shaojie Li, Xianyun Sun

**Affiliations:** a State Key Laboratory of Mycology, Institute of Microbiology, Chinese Academy of Sciences, Beijing, China; b College of Life Sciences, University of Chinese Academy of Sciences, Beijing, China; University of Guelph

**Keywords:** azole resistance, kinase, STK-17, *Neurospora crassa*, *Fusarium verticilioides*, sterol biosynthesis, azole accumulation

## Abstract

The emergence of antifungal resistance, especially to the most widely used azole class of ergosterol biosynthesis inhibitors, makes fungal infections difficult to treat in clinics and agriculture. When exposed to azoles, fungi can make adaptive responses to alleviate azole toxicity and produce azole tolerance. However, except for azole efflux pumps and ergosterol biosynthesis genes, the role of most azole responsive genes in azole resistance is unknown. In this study, STK-17, whose transcription is upregulated by azoles, was characterized as a novel kinase that is required for azole resistance. Deletion or dysfunction of STK-17 led to azole hypersensitivity in Neurospora crassa and to other ergosterol biosynthesis inhibitors such as amorolfine, terbinafine, and amphotericin B, but not fatty acid and ceramide biosynthesis inhibitors. STK-17 was also required for oxidative stress resistance, but this was not connected to azole resistance. RNA-seq results showed that *stk-17* deletion affected the basal expression and the response to ketoconazole of some membrane protein genes, indicating functional association of STK-17 with the membrane. Notably, deletion of *stk-17* affected the normal response to azoles of *erg* genes, including the azole target-encoding gene *erg11,* and *erg2*, *erg6*, and *erg24*, and led to abnormal accumulation of sterols in the presence of azoles. HPLC-MS/MS analysis revealed increased intracellular azole accumulation in the *stk-17* mutant, possibly due to enhanced azole influx and reduced azole efflux that was independent of the major efflux pump CDR4. Importantly, STK-17 was widely distributed and functionally conserved among fungi, thus providing a potential antifungal target.

**IMPORTANCE** Antifungal resistance is increasing worldwide, especially to the most widely used azole class of ergosterol biosynthesis inhibitors, making control of fungal infections more challenging. A lot of effort has been expended in elucidating the mechanism of azole resistance and revealing potential antifungal targets. In this study, by analyzing azole-responsive genes in Neurospora crassa, we discovered STK-17, a novel kinase, that is required for azole resistance in several types of fungi. It has a role in regulating membrane homeostasis, responses to azole by ergosterol biosynthesis genes and azole accumulation, thus, deepening our understanding on the mechanism of azole stress response. Additionally, STK-17 is conserved among fungi and plays important roles in fungal development and stress resistance. Kinase inhibitors are broadly used for treating diseases, and our study pinpoints a potential drug target for antifungal development.

## INTRODUCTION

Fungal infections have large impacts on human health and agriculture (1). In spite of decades of research, only limited classes of antifungal drugs and fungicides are currently available (2, 3). Azoles, which inhibit ergosterol biosynthesis by directly binding to sterol 14α-demethylase, are the most widely used antifungals in clinics and agriculture. However, numerous fungal pathogens have developed azole resistance and this is now a serious problem leading to therapy failure (2–4). Given the difficulties in developing antifungal drugs, increasing our understanding of how fungi neutralize antifungal compounds such as azoles would be helpful for maximizing their efficacy and to identify new antifungal targets.

When challenged by azole stress, fungi can make adaptive responses by changing expression of relevant genes across the genome, including those involved in ergosterol biosynthesis, efflux pumps, cell wall maintenance, lipid and amino acid metabolism, cell cycle regulation, and signal transduction (5–9). These changes allow fungi to quickly adjust their physiological state and maximize survival against azole exposure (10). Fungi lacking these responses are extremely sensitive to azoles (11). Deletions or mutation in transcription factors that regulate azole stress response, such as Pdr1/3p and Upc2p in yeast, Tac1p and Upc2p in Candida albicans, AtrR and SrbA in Aspergillus fumigatus, and CCG-8, ADS-4, CSP-1 in Neurospora crassa (5, 6, 12–18), cause alterations in azole sensitivity. Among these azole-responsive genes, the well-studied are those encoding the azole efflux pumps and sterol 14α-demethylase, as well as other ergosterol biosynthesis enzymes (2). Mutations leading to over-expression of azole efflux pumps, and those affecting sterol 14α-demethylase and enzymes involved in ergosterol biosynthesis, are frequently detected in azole-resistant isolates (2–4). Thus, genes required for reducing azole stress are often transcriptionally induced by azoles and this response can eventually result in resistance.

However, except for genes encoding azole efflux pumps and ergosterol biosynthesis enzymes, the roles of most of the azole-responsive genes in azole resistance have not been well characterized. One interesting and potentially important observation is that expression of some kinase genes is affected by azoles. Kinases, which regulate protein functions by phosphorylation, play important roles in adapting to stresses in fungi (19), and because they are more conserved than transcription factors, they may be useful as potential antifungal targets (19, 20). Some kinase genes were found to be co-regulated with azole efflux pumps and ergosterol biosynthesis genes, and were proposed to function in regulating plasma membrane function (21). Therefore, to gain a more comprehensive understanding of azole resistance and hopefully reveal more potential drug targets, this study focused on the functional characterization of the azole-responsive kinase genes.

We examined a previously reported azole-responsive kinase, STK-17, from N. crassa (16) and found that it could confer azole resistance in N. crassa and Fusarium *verticilioides*. STK-17 has a positive role in azole resistance through regulating membrane and ergosterol homeostasis and intracellular azole accumulation. Also, the azole resistance mediated by STK-17 is conserved among fungi and, thus, is a potential target for antifungal drug development.

## RESULTS

### Kinase STK-17 confers azole sensitivity in N. crassa.

**FIG 1:**
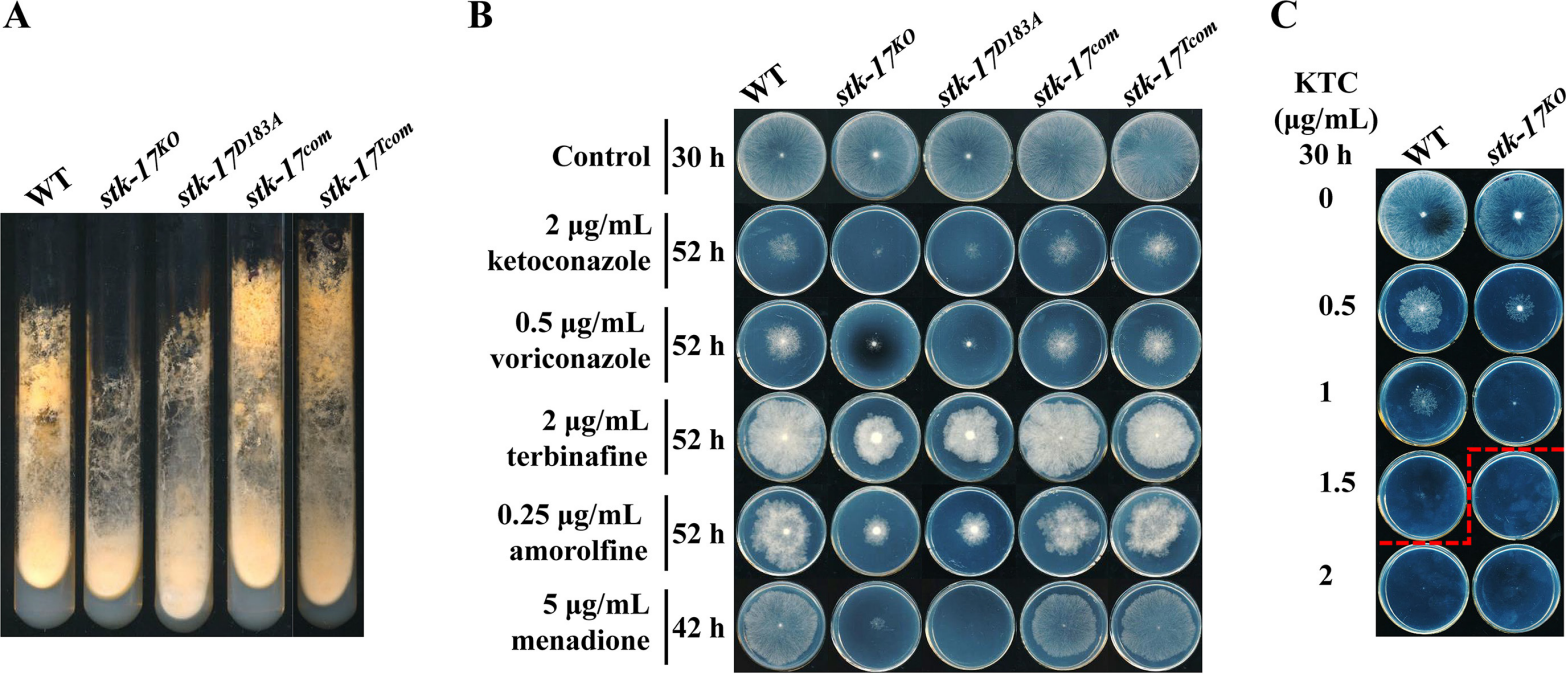
STK-17 is required for conidiation and antifungal resistance in Neurospora crassa. (A) STK-17 is required for conidiation. Conidia of WT, *stk-17^KO^* strain, *stk-17^D183A^* strain, *stk-17^com^* strain, and *stk-17^tcom^* strain were inoculated on Vogel’s slants and grown at 28°C for 7 days. (B) Sensitivity test of WT, *stk-17^KO^* strain, *stk-17^D183A^* strain, *stk-17^com^* strain, and *stk-17^tcom^* strain to various ergosterol biosynthesis inhibitors and oxidative stress inducer, menadione. Conidia suspensions (2 μL of 2 × 10^6^ conidia/mL) for each strain were inoculated on Vogel’s plates with or without indicated chemicals. The plates were incubated at 28°C and the colonies were documented at indicated time point. (C) Sensitivity test of WT and *stk-17^KO^* strain to various concentrations of ketoconazole (KTC). Conidia suspensions (2 μL of 2 × 10^6^/mL) for each strain were inoculated on Vogel’s plates with 0, 0.5, 1, 1.5, or 2 μg/mL KTC. After incubation at 28°C for 30 h, the growth phenotype of each strain on each plate was documented. Red dashed line indicates complete inhibition by KTC.

In our previous study we found that NCU04990, which encodes kinase STK-17, increased its transcript level under ketoconazole (KTC) stress (16). However, the function of STK-17 under azole stress is unknown. To address this question, the *stk-17* deletion mutant (*stk-17^KO^*) was first phenotypically characterized. As shown in Fig. 1A and B, although deletion of *stk-17* in N. crassa resulted in reduced conidial production on slants as previously reported (22), the colony growth of *stk-17*^KO^ on agar plates was similar (95% ± 5%) to that of wild-type (WT) (Fig. 1B). However, on plates containing 2 μg/mL KTC, the colony size of *stk-17^KO^* was only 30% ± 3% of WT after 52-h growth (Fig. 1B), indicating that the *stk-17^KO^* strain was hypersensitive to KTC. The *stk-17^KO^* strain was also hypersensitive to several other antifungal azoles, including voriconazole (Fig. 1B), fluconazole, and itraconazole (Fig. S1A). KTC was used as the primary azole drug in subsequent experiments with N. crassa.

To examine if the azole hypersensitivity phenotype of this mutant was concentration dependent, the growth of *stk-17^KO^* at various concentrations (0 to 2 μg/mL) of KTC was compared with that of WT. As shown in Fig. 1C, *stk-17^KO^* was more inhibited than WT at each concentration. The growth of *stk-17^KO^* was completely inhibited by 1.5 μg/mL KTC, while complete inhibition of WT required 2 μg/mL KTC. This result was further verified by determination of MIC of KTC, which turned out to be 1.5 μg/mL for *stk-17^KO^* and 2.0 μg/mL for WT.

To confirm that the azole hypersensitivity in the mutant was caused by *stk-17* deletion, the *stk-17^KO^* strain was complemented with its own coding sequence flanked by the native promoter and terminator. The complemented strain (*stk-17^com^*) grew normally on slants and produced abundant conidia similar to WT (Fig. 1A) and regained WT sensitivity to azoles (Fig. 1B), confirming the role of STK-17 in conferring basal azole resistance and conidiation.

To determine if the kinase activity of STK-17 was responsible for its function, a sequence expressing a 5×myc-6×his-tagged kinase-dead (D183A) version of the kinase (*stk-17^D183A^*) under the control of its own promoter and terminator was introduced into the *stk-17^KO^* strain, and a control strain expression a 5×myc-6×his-tagged non-mutated kinase (*stk-17^Tcom^*) was also generated. The re-introduction of *stk-17* in the obtained strains was verified by qRT-PCR (Fig. S2A). And we noticed that its expression is 19-fold higher in the *stk-17*^D183A^ than that in the WT and *stk-17^Tcom^* strain (Fig. S2A). The *stk-17^Tcom^* strain displayed KTC susceptibility and conidial production similar to WT and *stk-17^com^*, while the *stk-17^D183A^* strain was similar to *stk-17^KO^* (Fig. 1A and B). Thus, the STK-17 kinase activity is required for basal resistance to azole antifungals, as well as normal conidial development. Surprisingly, the protein of STK-17 could not be detected in above-mentioned strains (Fig. S2B), indicating that STK-17 may be a low abundant protein.

### STK-17 is required for basal resistance to ergosterol biosynthesis inhibitors and oxidative stress.

In addition to azoles, the *stk-17^KO^* strain and the *stk-17^D183A^* strain displayed greater sensitivity to terbinafine and amorolfine (Fig. 1B), while the *stk-17^com^* strain and the *stk-17^Tcom^* strain exhibited sensitivity to these two drugs similar to WT. Terbinafine targets squalene epoxidase ERG1, an enzyme upstream of ERG11, while amorolfine targets sterol C8-C7 isomerase and sterol reductase (ERG2 and ERG24, respectively), two enzymes downstream of ERG11 in the ergosterol biosynthetic pathway. Deletion of *stk-17* also resulted in hypersensitive to amphotericin B, which directly binds to ergosterol in the membrane (Fig. S1B). Thus, deletion of *stk-17* or functional loss of STK-17 kinase activity generally impaired the basal resistance to ergosterol biosynthesis inhibitors with different antifungal targets.

Because sterols are important membrane lipids, we then tested whether STK-17 function was also associated with other membrane lipids by examining the drug sensitivity of the *stk-17^KO^* strain to two other inhibitors of membrane lipid biosynthesis, cerulenin and myriocin. Cerulenin is an antifungal antibiotic that inhibits fatty acid biosynthesis, while myriocin is an inhibitor of sphingolipid biosynthesis. Both compounds were previously used in N. crassa to inhibit fatty acid and ceramide biosynthesis, respectively (23, 24). Under treatment with each antifungal, the growth of both WT and the *stk-17^KO^* strain was inhibited by at least 50% compared with control. However, no difference in susceptibility to each of these antifungals was found between the two strains (Fig. S1C).

We also tested the sensitivity of the *stk-17^KO^* strain to other stresses, including osmotic stress induced by NaCl, cell wall stress induced by caspofungin, oxidative stress induced by menadione, and cell membrane stress induced by SDS. The *stk-17^KO^* strain and the *stk-17^D183A^* strain, but not the *stk-17^com^* strain and the *stk-17^Tcom^* strain, displayed greater sensitivity to menadione (Fig. 1B), indicating a role of STK-17 in the oxidative stress response. However, deletion of *stk-17* did not affect the sensitivity to 1 M NaCl, 1 μg/mL caspofungin, or 0.005% SDS (Fig. S1DEF). This suggests that STK-17 is required for basal resistance to ergosterol biosynthesis inhibitors, as well as oxidative stress.

### Oxidative stress is not a mediator of increased azole sensitivity in the *stk-17^KO^* strain.

**FIG 2:**
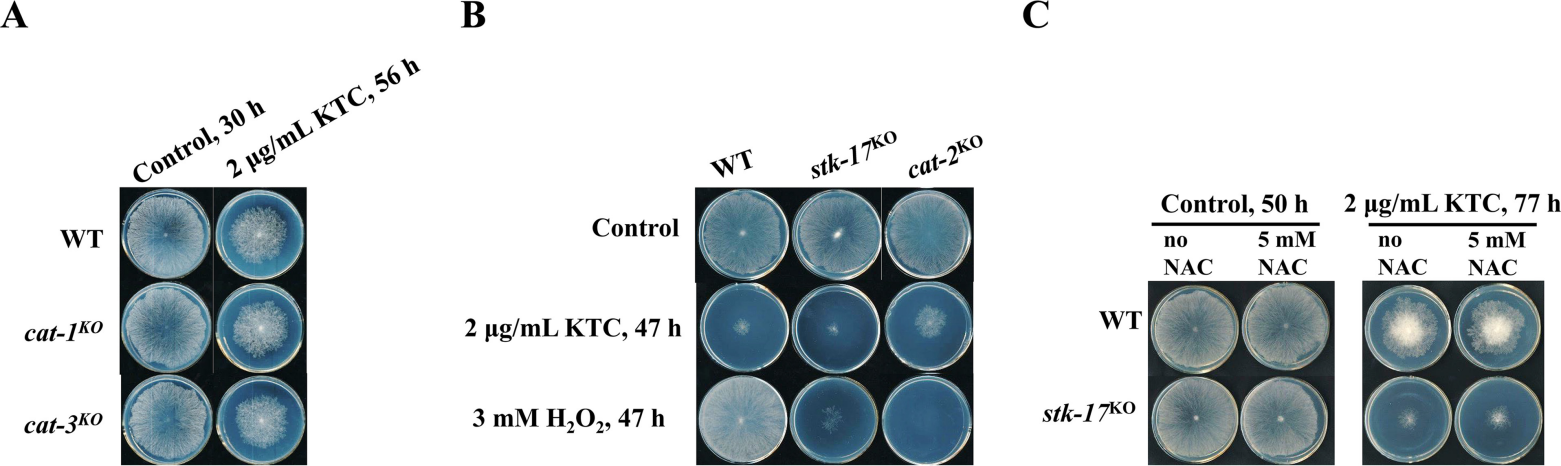
Oxidative stress is not a mediator of azole hypersensitivity of *stk-17^KO^* strain. (A) Sensitivity test of WT, *cat-1^KO^* strain, and *cat-3^KO^* to ketoconazole (KTC). (B) Sensitivity test of WT and *cat-2^KO^* strain to KTC and H_2_O_2_. (C) N-acetyl-cysteine (NAC) did not attenuate the sensitivity of WT and *stk-17^KO^* strain to KTC. Conidia suspensions (2 μL of 2 × 10^6^ conidia/mL) for each strain were inoculated on Vogel’s plates with or without indicated chemicals. The plates were incubated at 28°C and the colonies were documented at the indicated time points.

Deletion of *stk-17* increased sensitivity of N. crassa to both azole stress and oxidative stress (Fig. 1B). Because azoles like miconazole exert toxicity by inducing oxidative stress (25, 26), it is possible that the azole hypersensitivity of the *stk-17^KO^* strain resulted from the oxidative stress induced by azoles. If that is the case, genes involved in scavenging ROS would be induced by azoles and their deletion mutants would be hypersensitive to azoles. In our previous results, we found that only *cat-2*, a catalase gene, was dramatically induced by ketoconazole (5, 6). Thus, we examined the sensitivity of *cat-2*^*KO*^ to azoles, as well as mutants of other two catalase genes *cat-1* and *cat-3*. However, deletion of *cat-1* or *cat-3* did not result in hypersensitive to azoles (Fig. 2A). Interestingly, deletion of *cat-2* led to resistance to ketoconazole, although this mutant was hypersensitive to H_2_O_2_ (Fig. 2B). To further explore the putative relationship between azole hypersensitivity and sensitivity to oxidative stress in the *stk-17^KO^* strain, the antioxidant N-acetyl cysteine (NAC) was added to the medium for the azole sensitivity test. At a concentration of 5 mM that did not affect the growth of either WT or the *stk-17^KO^* strain, NAC had no effect on azole sensitivity of WT or the *stk-17^KO^* strain (Fig. 2C). Above results mean that ketoconazole toxicity may be independent of oxidative stress in N. crassa. And the hypersensitivity to oxidative stress and to azoles in the *stk-17^KO^* strain may be independent and caused by different mechanisms.

### Genome-wide transcriptional responses to *stk-17* deletion.

To gain insights into the molecular basis by which STK-17 regulates basal azole resistance, we determined STK-17-dependent gene expression using RNA-sequencing in the presence or absence of azoles. Transcripts of a total of 9,990 genes were detected and 6,713 genes were used for subsequent identification of differentially expressed genes after removing genes with FPKM <5 in all samples.

Genome-wide transcriptional change was induced by deletion of *stk-17*. In the absence of KTC treatment, expression of 181 genes (139 upregulated and 42 downregulated) were concomitantly affected by the *stk-17* deletion compared with WT and the *stk-17^com^* strain (Fig. S3A, Data set S1 Sheet 1). With KTC treatment, 776 genes were affected by *stk-17* deletion, including 522 upregulated and 254 downregulated genes (Fig. S3D, Data set S1 Sheet 2). Among these genes, only 123 were affected by *stk-17* deletion under both KTC and non-KTC conditions (Data set S1 Sheet1, lines in bold).

Because fungi can adapt to azole stress by modifying gene expression, further analysis was conducted to determine the role of STK-17 in the azole stress response. In both the WT and the complementation strain, 1,450 genes were responsive to KTC, including 851 upregulated genes and 599 downregulated genes (Data set S1 Sheet 3). These KTC-responsive genes were then compared with the 776 genes affected by KTC treatment of the *stk-17* deletion strain by Venn analysis. The expression of 282 KTC-upregulated genes was higher in the *stk-17^KO^* strain treated with KTC and expression of 108 KTC-downregulated genes was lower in *stk-17^KO^* strain treated with KTC (Fig. S3E). Among these genes, the gene encoding the transcription factor ADS-4, which is required for the azole stress responses (5), showed elevated expression in the *stk-17^KO^* strain under KTC treatment, together with its regulated genes *abcB* (NCU03773), *rta2* (NCU05209), *mnn4* (NCU3213), and *mmt2* (NCU07879) (Data set S1 Sheet 2, lines marked as bold). Because the *stk-17^KO^* strain was hypersensitive to azole, these genes in the *stk-17^KO^* cell might need to be upregulated to higher levels than WT to overcome azole stress. Expression of 343 genes (221 upregulated and 122 downregulated) were only changed under KTC due to *stk-17* deletion (Fig. S3E, Data set S1 Sheet 2). Several of them were affected under the non-KTC condition, but the majority of them showed responses to KTC in the *stk-17^KO^* strain (Data set S1 Sheet 2). Interestingly, many of these genes were KTC-responsive in our previous study in N. crassa, in which KTC concentration was higher and the treatment time was longer (6). Thus, like ADS-4, these genes may also need to be upregulated to higher levels to overcome azole stress. Only 43 genes showed reduced response to KTC in the *stk-17^KO^* strain as revealed by Venn analysis (Fig. S3E, Data set S1 Sheet 2), among which 24 genes were KTC-upregulated but less induced in the *stk-17^KO^* strain by KTC and 19 genes were KTC downregulated but less repressed in the *stk-17^KO^* strain by KTC. Thus, deletion of *stk-17* affected transcriptional responses of certain genes to azole stress.

### STK-17 regulates expression of genes encoding transmembrane proteins.

**FIG 3:**
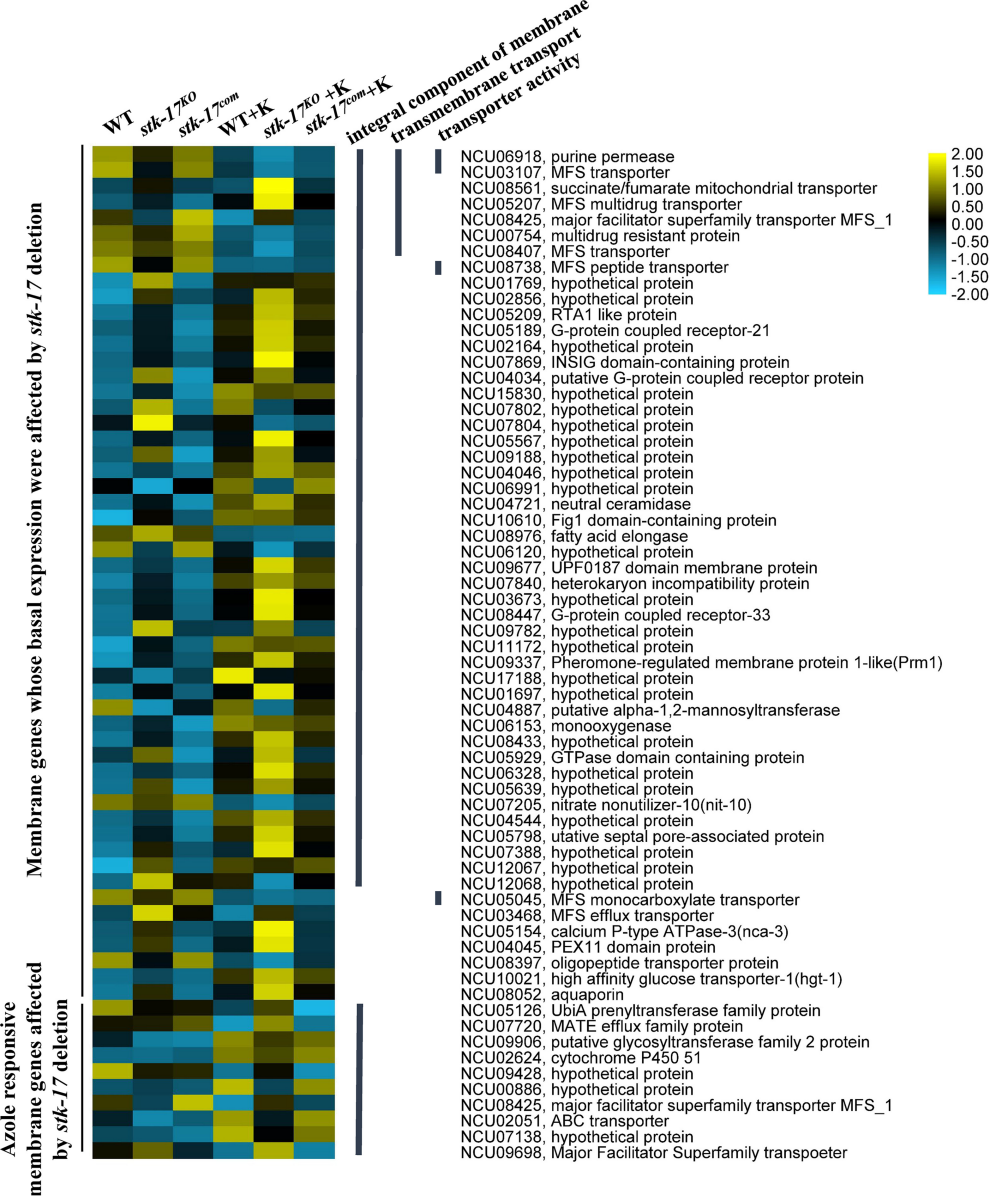
STK-17 regulates the expression of membrane genes. Heatmap showing expression of genes in WT, *stk-17^KO^* strain, and *stk-17^com^* strain under ketoconazole treated (+K) and non-treated condition. Genes selected were enriched membrane genes whose basal expression or responses to azoles were affected by *stk-17* deletion. The average FPKM values of each gene in each sample were used for heatmap construction after log transformation and scaling. The heatmap was constructed, visualized, and edited with TBtools. The thick lines in the middle of the figure indicate the GO terms of each gene.

To further explore the biological processes affected by *stk-17* deletion, the 181 genes affected by *stk-17* deletion under non-KTC condition and 43 KTC responsive genes affected by *stk-17* deletion under KTC condition were subjected to enrichment analysis. Functional categories enrichment analysis of the 181 genes revealed that 54 of them were enriched in the category of transmembrane and transmembrane helix (Fig. S2B, Data set S1 Sheet 1), and most of them (41 out of 54) were upregulated (Fig. 3, Data set S1 Sheet 1), indicating that deletion of *stk-17* affected the expression of membrane proteins. Moreover, only three GO terms, integral component of membrane (47 genes), transmembrane transport (7 genes) and transporter activity (4 genes), were enriched with *P* values <0.05 (Fig. S3C, Fig. 3). Among these genes, transcription of the genes encoding INSIG domain-containing protein (NCU07869), fatty acid elongase (NCU08976), and neutral ceramidase (NCU04721) were increased 2.4-, 1.7-, and 1.7-fold, respectively (Fig. 3, Data set S1 Sheet 1). The INSIG domain is involved in sterol homeostasis, while fatty acid elongase and neutral ceramidase are required for membrane lipid biosynthesis and metabolism (17, 27, 28). In addition, the majority of the upregulated transmembrane genes, such as NCU07869, NCU08976, NCU04721, NCU08052, NCU05639, and NCU05567, were KTC-responsive and their responses to KTC were enhanced in the *stk-17^KO^* strain (Fig. 3, Data set S1 Sheet 1), indicating a role of these genes in membrane homeostasis in response to azole treatment. Thus, the upregulation of these genes in the *stk-17^KO^* strain suggests that normal membrane function may be impaired in the *stk-17^KO^* strain.

For the 43 genes, only the term “integral component of membrane” was enriched in GO enrichment analysis (10 genes, Fig. S3F, Fig. 3) and only the term “steroid biosynthesis” was enriched in the KEGG enrichment analysis (two genes, Fig. S3G). The enriched steroid biosynthesis genes were *erg11* and *erg6*, whose expression in *stk-17^KO^* strain under KTC was only 66% and 64% that of wild-type level, respectively (Data set S1 Sheet 2); therefore, STK-17 may be involved in normal membrane function and responses to azole by ergosterol biosynthesis genes.

### STK-17 is required for the responses of ergosterol biosynthesis genes to azoles.

**FIG 4:**
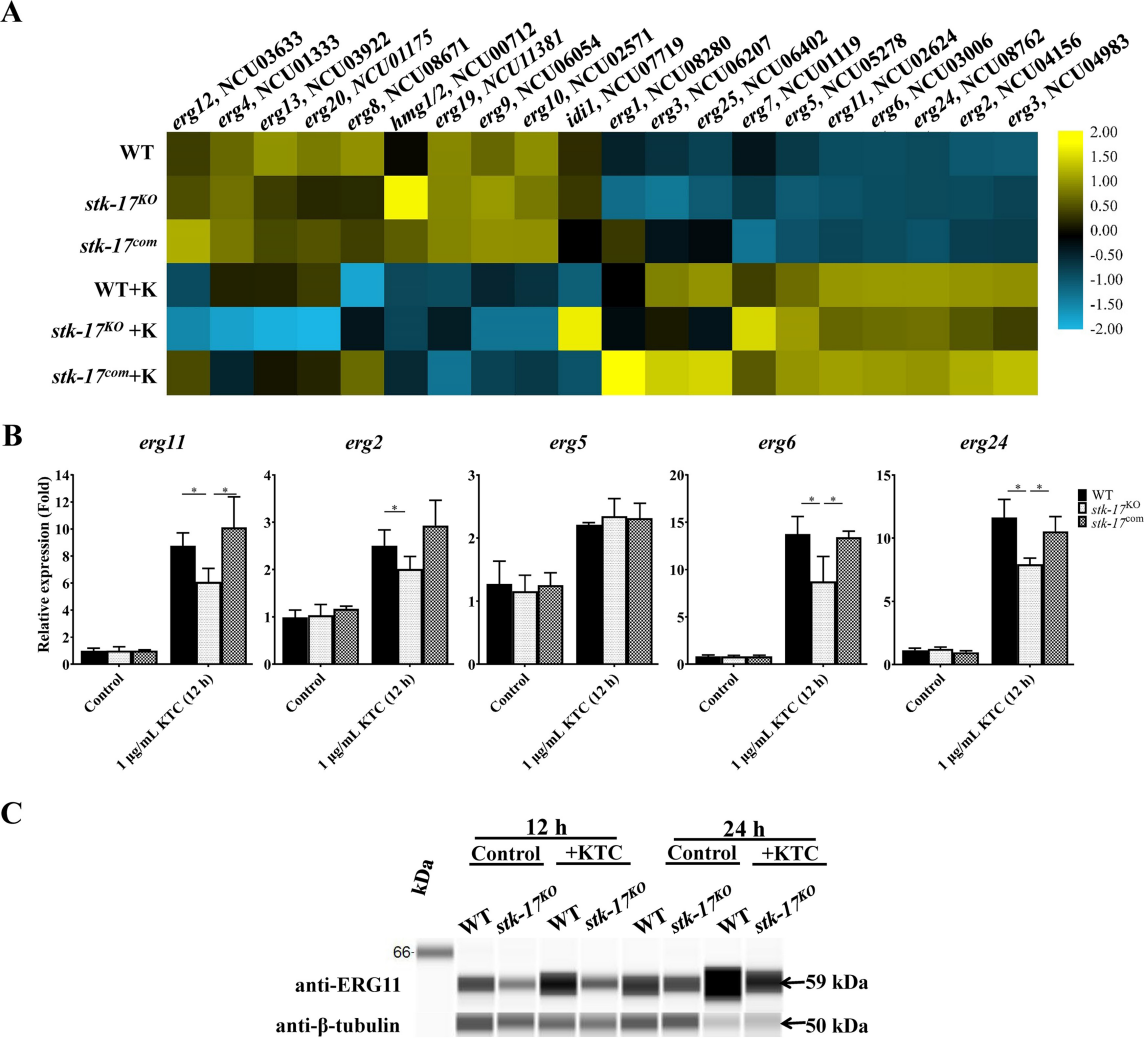
STK-17 is required for ergosterol biosynthesis under azole stress. (A) Heatmap presentation of the expression of *erg* genes in WT, the *stk-17^KO^* strain, and the *stk-17^com^* strain under ketoconazole (KTC) treated (+K) and non-treated conditions. The average FPKM values of each gene in each sample were used for heatmap construction after log transformation and scaling. The heatmap was constructed, visualized, and edited with TBtools. (B) STK-17 is required for the responses of *erg* genes to KTC. After grown in liquid Vogel’s medium for 13.5 h, the mycelium of WT, the *stk-17^KO^* strain, and the *stk-17^com^* strain were treated with 1 μg/mL KTC for an additional 12 h. The transcript levels of *erg11* and *erg2*, *erg5*, *erg6*, and *erg24* were measured by quantitative real-time PCR (qRT-PCR), and the expression was calculated using the 2^-ΔΔCt^ method normalized to β-tubulin. The results presented here are means of three biological replicates, and the significant levels were calculated by *t* test and marked as *, *P* < 0.05. (C) Detection of ERG11 protein by Western blotting in WT and the *stk-17^KO^* strain treated with or without KTC. After grown in liquid Vogel’s medium for 13.5 h, the mycelium of WT and the *stk-17^KO^* strain were then treated with KTC for an additional 12 h or 24 h. The protein levels of ERG11 were detected by using an ERG11 specific antiserum. The protein levels of β-tubulin were used as a loading control.

The RNA-seq analysis revealed that deletion of *stk-17* affected the responses of *erg11* and *erg6* to azoles. We checked the expression of other *erg* genes and found that their responses to KTC were also affected by *stk-17* deletion, including *erg2* (NCU04156), *erg3* (NCU06207, NCU04983), *erg24* (NCU08762), and *erg25* (NCU06402) (Fig. 4A), although the fold change was <1.5. Thus, STK-17 globally regulates ergosterol biosynthesis in response to azole exposure. To confirm the above results, we measured the expression of *erg11* in *stk-17^KO^* strain, as well as WT, treated with or without azoles, by qRT-PCR. In WT, the transcript level of *erg11* increased 9-fold after incubation with 1 μg/mL KTC for 12 h (Fig. 4B). The basal expression of *erg11* was not affected by *stk-17* deletion, but it increased only 6-fold in response to KTC (Fig. 4B). The response of *erg11* to KTC in *stk-17^com^* strain was similar to WT, confirming the requirement of STK-17 in maintaining the proper transcriptional response of *erg11* to azoles. The response of *erg11* to treatment with different concentrations of KTC or for a longer period of 24 h was consistently affected by *stk-17* deletion (Fig. S4A and B). We also measured the ERG11 protein level in WT and the *stk-17^KO^* strain using an ERG11-specific antibody. As expected, the ERG11 protein level increased with KTC but was attenuated by *stk-17* deletion (Fig. 4C). Thus, STK-17 regulates the response of *erg11* to azole at both the transcription and protein level. The expression of other *erg* genes was also determined by qRT-PCR. In N. crassa, *erg2* (NCU04156), *erg5* (NCU05278), *erg6* (NCU03006), and *erg24* (NCU08762) all showed a transcriptional response to azoles and were required for azole resistance (29); therefore, we only tested these genes. Similar to *erg11*, the responses of *erg2*, *erg6* and *erg24* (but not *erg5*) to azoles were attenuated by *stk-17* deletion (Fig. 4B, Fig. S4A), in agreement with the RNA-seq results. Thus, STK-17 is required for the responses of other *erg* genes to azoles.

Next, we compared the sterol profiles of WT and the *stk-17^KO^* strain treated with or without KTC. Under non-KTC conditions, the WT sterol profile and that of the *stk-17^KO^* strain were similar. As previously reported for N. crassa (16, 30), KTC treatment led to reduction in ergosterol level and accumulation of sterol intermediates such as lanosterol, eburicol, and a toxic sterol, 14α-methyl-3,6-diol (Table 1), as revealed by two independent analyses. However, deletion of *stk-17* altered the sterol profiles under KTC treatment. In WT, KTC treatment reduced ergosterol levels to 55% and 40%, respectively, in two independent analysis, while the decrease was 15% more in the *stk-17^KO^* strain than in WT (Table 1). In addition, the concentration of lanosterol and eburicol under KTC treatment in the *stk-17^KO^* strain was higher than that in wild type (Table 1). We did not find a higher level of 14α-methyl-3,6-diol in the *stk-17^KO^* strain (Table 1), although it is a by-product from lanosterol and eburicol through an alternative biosynthetic pathway, indicating its conversion pathway was affected. Unexpectedly, an unknown sterol with a UV absorbance peak at 280 nm and *m/z* of 377 and 393 was found to be 4-fold higher in the *stk-17^KO^* strain (Table 1 and Fig. S5). Thus, STK-17 is required for sterol homeostasis under azole stress.

**TABLE 1:** Sterol contents in N. crassa WT and *stk-17^KO^* strain treated with or without ketoconazole^*a*^

Sample		Normalized relative amount				
		Ergosterol (RT = 7.43 m/z = 379)	Lanosterol (RT = 8.8 m/z = 409)	Eburicol (RT = 9.7 m/z = 423)	14α-methyl-3,6-diol (RT = 4.88 m/z = 411)	Unknown (RT = 4.135 m/z = 377)
First time	WT	1	1	1	NA	1
	*stk-17* ^KO^	0.936963293	1.4558808	0.5395822	NA	0.4183652
	WT-K	0.399318266	5.7413887	34.803441	1	0.5321747
	*stk-17*^KO^-K	0.261997774	12.311462	131.52387	0.994192623	8.4854378
Second time	WT	1	1	1	NA	1
	*stk-17* ^KO^	0.92234653	4.6967224	0.4599823	NA	0.0911043
	WT-K	0.549896763	20.015507	17.158954	1	0.6625718
	*stk-17*^KO^-K	0.406244225	22.815779	28.971442	1.09345252	3.426908

a WT, wild type; WT-K or *stk-17^KO^*-K, wild type or *stk-17^KO^* mutant treated with ketoconazole, respectively; RT, retention time; m/z, mass-to-charge ratio.

### Deletion of *stk-17* affects intracellular KTC accumulation independent of the azole efflux pump, CDR4.

**FIG 5:**
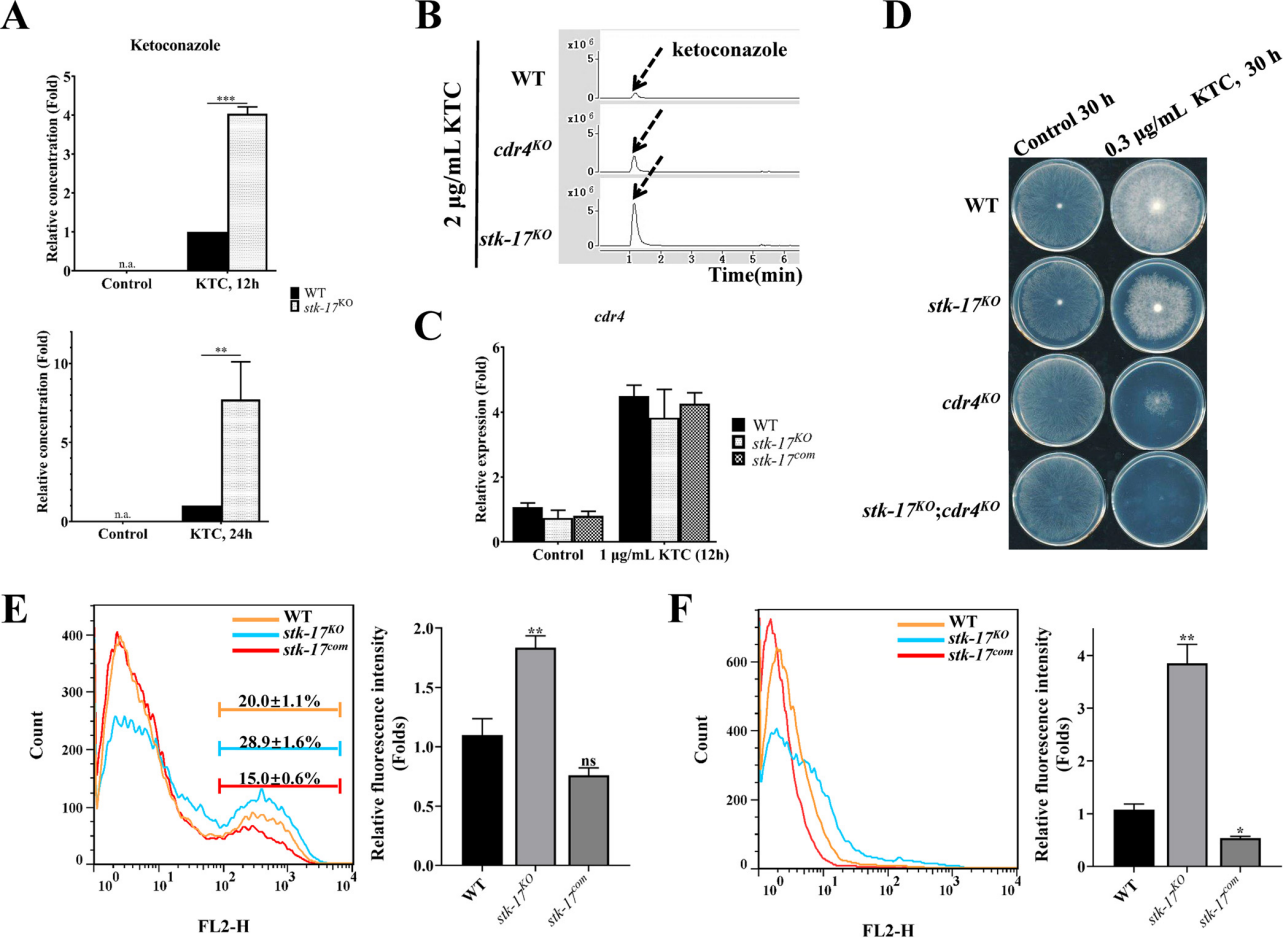
STK-17 regulates the intracellular accumulation of azoles. (A) Deletion of *stk-17* resulted in more accumulation of intracellular ketoconazole (KTC). (B) Differential accumulation of KTC in WT, *cdr4^KO^* strain, and the *stk-17^KO^* strain. (C) STK-17 did not regulate expression of azole efflux pump gene *cdr4*. After growing in liquid Vogel’s medium for 13.5 h, the mycelium of WT, the *stk-17^KO^* strain, the *stk-17^com^* strain, and/or the *cdr4^KO^* strain were treated with KTC for an additional 12 h or 24 h. The intracellular KTC (A and B) and total RNA (C) were then extracted and analyzed by HPLC-MS and qRT-PCR, respectively. The results presented here are means of three biological replicates, and the significance levels were calculated by *t* test and marked as *, *P* < 0.05; **, *P* < 0.01; or ***, *P* < 0.001. (D) Sensitivity test of WT, the *stk-17^KO^* strain, the *cdr4^KO^* strain, and the *stk-17^KO^*; *cdr4^KO^* strain to KTC. (E) Influx of rhodamine 6G in WT, *stk-17^KO^* strain, and *stk-17^com^* strain. Conidia of each strain were germinated in liquid Vogel’s medium for 3 h. Then the germinated conidia of indicated strains were incubated with rhodamine 6G in PBS without glucose for 1 h. After washing with PBS, 30,000 treated conidia were analyzed by flow cytometry. (F) Efflux of rhodamine 6G in WT, *stk-17^KO^* strain, and *stk-17^com^* strain. Treated conidia in (E) were then incubated with PBS with glucose for 40 min and analyzed by flow cytometry. Results of flow cytometry were analyzed by Image J.

Intracellular accumulation of KTC can be detected along with sterol profile analysis. After a 24-h treatment, the intracellular KTC in the *stk-17^KO^* strain was 7-fold higher than that in WT (Fig. 5A and B). Similar results were obtained for KTC accumulation after a 12-h treatment (Fig. 5A and B).

Azole accumulation is largely affected by efflux pump activity (2), and CDR4 is the primary azole efflux pump in N. crassa (30–32). To determine if CDR4-mediated azole efflux affected the accumulation of KTC in the *stk-17^KO^* strain, we compared the expression of *cdr4* in WT and the *stk-17^KO^* strain. Deletion of *stk-17* did not affect the basal expression of *cdr4* under non-treatment conditions (Fig. 5C). KTC treatment significantly upregulated *cdr4* expression 4-fold in WT and similar results were found in the *stk-17^KO^* strain (Fig. 5C), indicating that STK-17 do not regulates CDR4 transcriptionally. We also compared intracellular KTC in a *cdr4^KO^* strain with the *stk-17^KO^* strain. As reported before (30), after a 24-h KTC treatment, deletion of *cdr4* resulted in 2-fold higher accumulation of intracellular KTC compared to WT. However, *stk-17* deletion led to much higher KTC accumulation than either the WT or the *cdr4^KO^* strain (Fig. 5B). Above all, CDR4 is unlikely to contribute to azole accumulation in the *stk-17^KO^* strain. We further verified this conclusion by determining the azole sensitivity of the double-deletion strain lacking both *stk-17* and *cdr4*. The double mutant was much more sensitive to KTC than either single-deletion mutant (Fig. 5D), proving that CDR4 has little effect on KTC hypersensitivity in the *stk-17^KO^* strain.

In fungi, rhodamine 6G (R6G) influx/efflux is used to mimic azole influx and efflux (33). To explore how STK-17 regulates intracellular azole accumulation, we compared R6G influx/efflux in WT with that in the *stk-17^KO^* strain. The influx of R6G was detected under glucose-starved condition. After incubating the starved cells with R6G for 1 h, the R6G fluorescence signal was detected in the geminated conidia of each strain (Fig. 5E). The percentage of conidia with high fluorescence intensity was higher in the *stk-17^KO^* strain compared with WT and *stk-17^com^* strain (Fig. 5E). The mean fluorescence intensity was 1.8-fold higher in the *stk-17^KO^* strain than that in WT and the *stk-17^com^* strain (Fig. 5E). Therefore, the *stk-17* deletion enhanced drug uptake. To determine R6G efflux, R6G incubated germinated conidia mentioned above were then incubated in PBS with glucose for 40 min. This treatment led to the loss of the high fluorescence intensity peak (Fig. 5E and F), indicating R6G efflux; however, there was a peak shift in the *stk-17^KO^* strain relative to WT and the *stk-17^com^* strain (Fig. 5F). The mean fluorescence intensity was 3.8-fold higher in the *stk-17^KO^* strain (Fig. 5F), indicating that drug efflux may also be mediated by STK-17. In summary, STK-17 may regulate azole accumulation by modulating both azole influx and efflux independent of the major azole efflux pump, CDR4.

### STK-17 has no interaction with known transcription factors that regulate azole stress response.

Because STK-17 affects sterol homeostasis, we examined if it acted through regulation of some known transcription factors. The function of sterol regulatory element binding proteins (SREBPs), the major regulator of ergosterol homeostasis and azole resistance in fungi (30), was first examined. However, deletion of SREBP homolog encoding gene *sah-2*, as well as the gene encoding rhomboid protease RBD-2 that is required for the activation of SREBP (30) in N. crassa did not result in azole hypersensitivity (Fig. S6AB). AtrR is another important transcription factor regulating ergosterol biosynthesis in Aspergillus fumigatus (15). Function of this transcription factor was also examined. However, deletion of *atrR* (NCU01478) in N. crassa did not change the sensitivity to ketoconazole (Fig. S6C). These results may indicate that STK-17 regulates ergosterol biosynthesis through some other genes.

Because deletion of *ccg-8* in N. crassa also resulted in azole accumulation and attenuated responses of *erg* genes to azoles similar to what was seen with the *stk-17^KO^* strain (6, 31), we examined the relationship between STK-17 and the transcription factor CCG-8. However, the double-deletion mutant of *stk-17* and *ccg-8* was more sensitive than either of the single-deletion mutants (Fig. S6D), indicating a weak connection between STK-17 and CCG-8.

### STK-17 genetically interacts with ERG11 under specific conditions.

**FIG 6:**
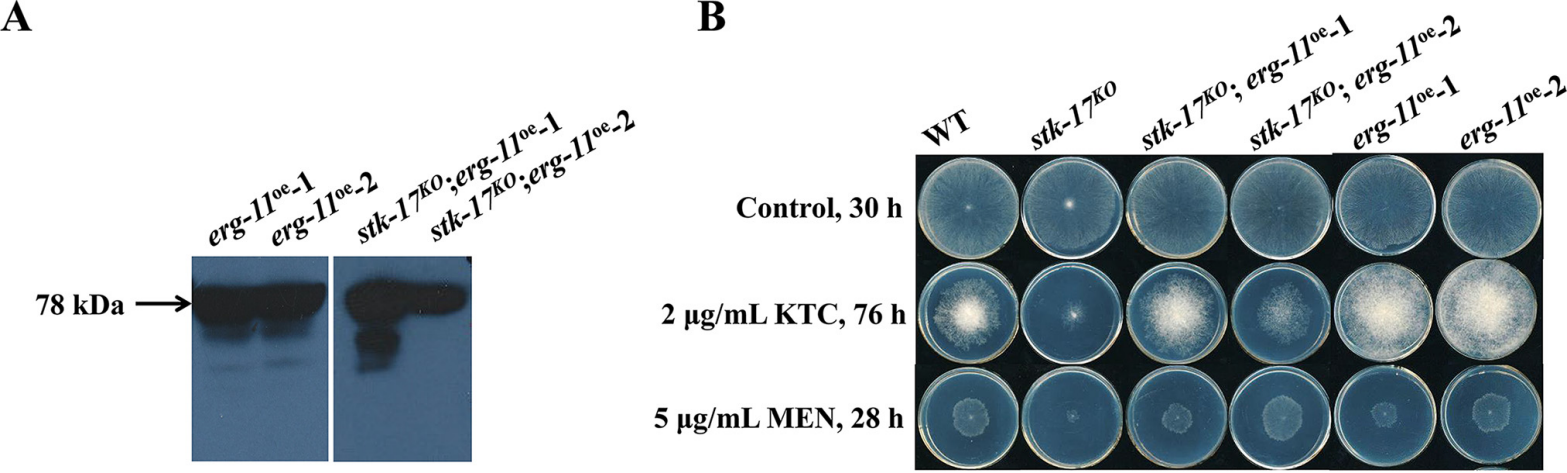
STK-17 has a genetic interaction with ERG11. (A) Western blotting detection of 5×myc-6×his tagged ERG11 in *erg11* overexpression strains using an anti-myc antiserum. The tagged ERG11 had a mass of 78 kDa (*arrow*). (B) Sensitivity test of WT, *stk-17^KO^* strain, and *erg11* overexpression strains to ketoconazole (KTC) and menadione (MEN). Conidia suspensions (2 μL of 2 × 10^6^/mL) for each strain were inoculated on Vogel’s plates with or without indicated chemicals. The plates were incubated at 28°C and the colonies were documented at the indicated time points.

Given that we found that STK-17 regulated the positive response of *erg11* to azoles, we further examined the relationship between STK-17 and ERG11 by constitutively overexpressing ERG11 in WT and the *stk-17^KO^* strain. Western blotting showed that ERG11 was successfully overexpressed in WT and the *stk-17^KO^* strain (Fig. 6A). Drug sensitivity test showed that overexpression of *erg11* alleviated the azole sensitivity of both WT and the *stk-17^KO^* strain, indicating that overexpression of *erg11* can contribute to azole sensitivity (Fig. 6B). Overexpression of *erg11* also restored the menadione sensitivity of the *stk-17^KO^* strain to WT level, but its overexpression in WT had little effect on menadione sensitivity (Fig. 6B). This indicates a genetic interaction between STK-17 and ERG11. However, overexpression of *erg11* had little effect on conidiation and sensitivity to amorolfine and terbinafine in both WT and the *stk-17^KO^* strain (Fig. S6E and F), meaning that the genetic interaction between STK-17 and ERG11 may be condition-specific.

### STK-17-mediated azole and oxidative stress sensitivity is functionally conserved among fungi.

**FIG 7:**
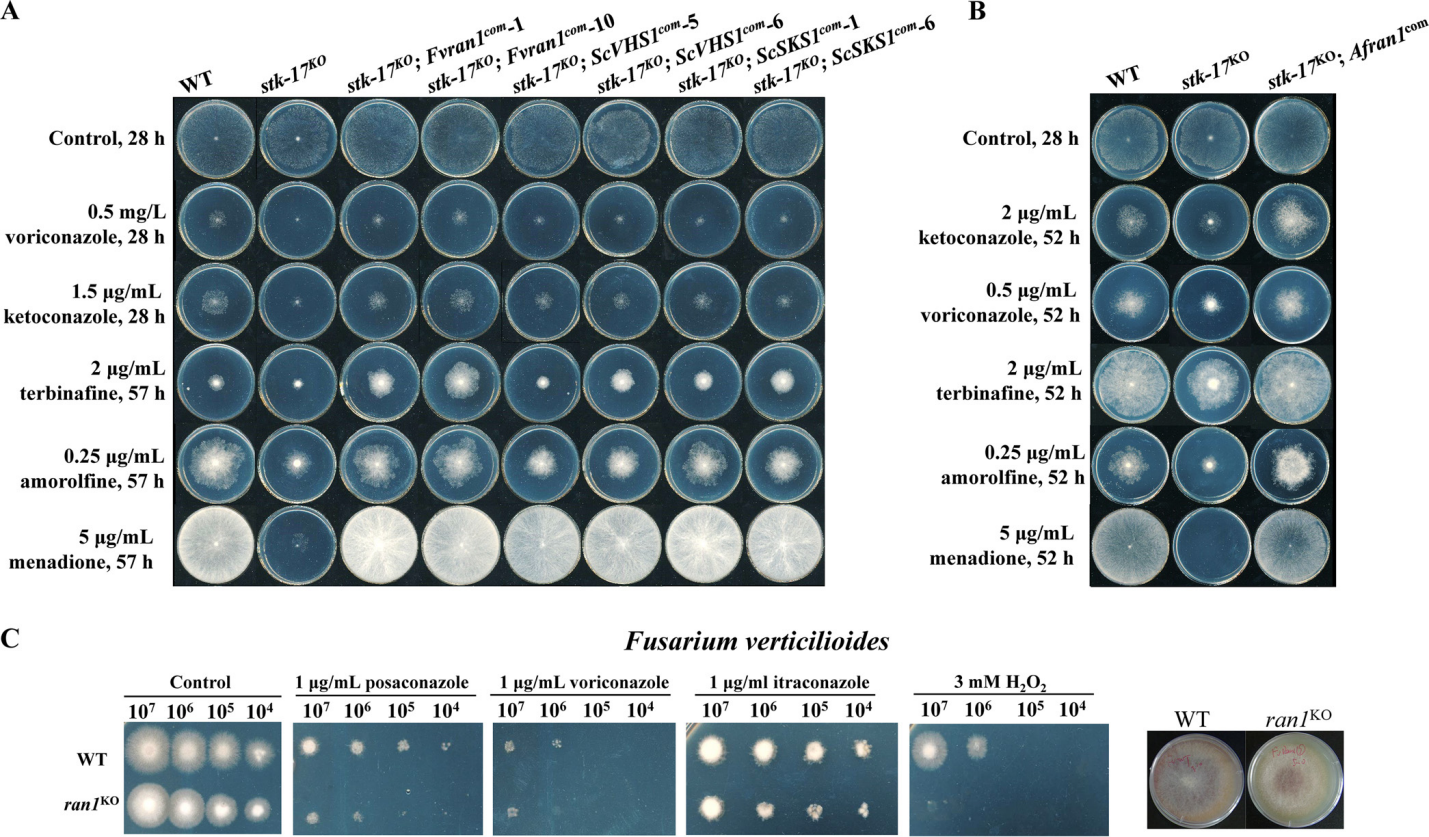
STK-17 protein is conserved among fungi. (A) and (B) The drug hypersensitive phenotypes of the *stk-17^KO^* strain can be restored by STK-17 homologs from Saccharomyces cerevisiae, Fusarium *verticilioides*, and Aspergillus fumigatus. *Fvran1*, (*stk-17* homolog of *F. verticilioides*); *ScVHS1*, and *ScSKS1* (*stk-17* homologs of S. cerevisiae); *Afran1* (*stk-17* homolog of A. fumigatus). (C) Deletion of STK-17 homolog Ran1 resulted in hypersensitive to azole and oxidative stress. Conidia of WT and *ran1^KO^* strain were serially diluted to 10^7^, 10^6^, 10^5^, and 10^4^ conidia/mL. The conidia suspensions (2 μL) were inoculated on plates with or without indicated chemicals. The plates were incubated at 28°C and the colonies were documented after 40 h.

The role of STK-17 in azole resistance in N. crassa is important for broadening our understanding of azole resistance in other fungi, especially pathogenic fungi. Protein sequence analysis and phylogenetic analysis indicate that STK-17 is widely distributed and conserved among fungi (Fig. S7A and B). To determine if STK-17 is functionally conserved in other fungi, we complemented the N. crassa *stk-17^KO^* strain with the STK-17 homologs, Vhs1p and Sks1p from Saccharomyces cerevisiae, Ran1 from A. fumigatus, and Ran1 from *F. veticilioides*, three phylogenetically divergent species. All these genes were controlled by the promoter of *stk-17*. Although S. cerevisiae Sks1p and Vhs1p only attenuated the defects in stress sensitivity but not conidiation, A. fumigatus Ran1 and *F. verticilioides* Ran1 restored all the phenotypes of the *stk-17^KO^* strain in drug sensitivity, oxidative stress, and conidiation (Fig. 7A and B, Fig. S7C and D), indicating a conserved function of STK-17 for stress resistance among fungi, especially filamentous fungi. We then deleted *ran1* in *F. verticilioides*, a plant pathogenic fungus. No defects in growth and conidiation were found in the deletion mutants; but, similar to the N. crassa *stk-17^KO^* strain, deletion of *ran1* in *F. verticilioides* resulted in hypersensitivity to posaconazole, virocoanzole, and itraconazole, as well as to the oxidative stress inducer H_2_O_2_ (Fig. 7C). Thus, STK-17 protein is functionally conserved in regulating azole and oxidative stress resistance among fungi.

## DISCUSSION

Azole resistance is a serious problem for clinicians in controlling fungal infections but also in agriculture. Fungi can make adaptive responses to azole stress by inducing expression of genes needed for azole resistance. However, the function of most azole responsive genes is poorly understood. In this study, a previously discovered azole-responsive kinase gene, *stk-17*, was functionally characterized and its deletion resulted in hypersensitive to azoles in N. crassa and *F. verticilioides.* Except for signal pathways, this is the first identified kinase that is required for azole resistance. Importantly, this kinase is conserved from yeast to filamentous fungi and has important roles in development and stress responses in N. crassa and other fungi (22, 34, 35), suggesting that it may be a promising drug target. Moreover, deletion of *stk-17* had broad effects on azole resistance, including ergosterol biosynthesis, azole uptake, and azole efflux, which are critical aspects of azole resistance, thus deepening our understanding on the regulation of the azole stress response.

STK-17 is an azole responsive kinase in N. crassa (6, 29). Moreover, STK-17 homologs in several other fungi have transcriptional responses to azoles and hypoxia (7, 8, 11, 36, 37). Hypoxia can inhibit ergosterol biosynthesis through oxygen-requiring steps (17). So, *stk-17* and its homologous genes may respond to perturbations in ergosterol biosynthesis. Our results clearly showed that *stk-17* deletion resulted in hypersensitive to azoles, chemicals that inhibit ergosterol biosynthesis in N. crassa and *F. verticilioides*. Similarly, chemical genomic analysis of S. cerevisiae revealed that mutants of the STK-17 homologs, Vhs1p and Sks1p, are hypersensitive to several azoles (38). Thus, the response of STK-17 to azole stress may contribute to azole resistance, and this could be directly associated with two mechanisms described below.

In several fungal species under azole stress, STK-17 and its homologs have been shown to be co-regulated with ergosterol biosynthesis. In C. albicans, Sha3p, a homolog of STK-17, was directly regulated by Upc2p, a major sterol biosynthesis regulator (39). In N. crassa, STK-17 was shown to be directly controlled under azole stress by CSP-1, a global transcriptional repressor, while CSP-1 was a negative regulator of ergosterol biosynthesis genes (16). This co-regulation of STK-17 and its homologs with ergosterol biosynthesis genes may imply a functional connection between each other. This hypothesis is further supported by our data showing that STK-17 genetically interacted with ERG11, an important enzyme in the ergosterol biosynthesis pathway. Our results also showed that deletion of *stk-17* altered the responses of ergosterol biosynthesis genes to azoles. This would partially explain the involvement of STK-17 in azole resistance. Azole inhibition resulted in ergosterol depletion and the accumulation of sterol intermediates (30). In response to changes in sterol profiles under azole stress, fungi could maintain sterol homeostasis by transcriptionally upregulating ergosterol biosynthesis genes, such as *erg2*, *erg6*, *erg5*, *erg24*, and *erg11*. To date, *erg11* and *erg5* were found to be beneficial to azole resistance (2, 29). Therefore, deletion of *stk-17* affected the response of ergosterol biosynthesis genes to azoles, resulting in azole-induced abnormal sterol profiles and hypersensitivity.

STK-17 also negatively regulates azole accumulation. To effectively inhibit ergosterol biosynthesis, azoles need to accumulate in the cell to a certain concentration, which is affected by both azole influx and efflux (2). Higher levels of azole accumulation resulted in azole hypersensitivity while lower accumulations led to resistance (30). Deleting *stk-17* affected both azole influx and efflux allowing azoles to accumulate in N. crassa and produce hypersensitivity. Azole import is mediated by unknown proteins (2). CDR4 is the major ABC azole transporter in N. crassa, but CDR4 is not regulated by STK-17 (32), suggesting the existence of other mechanisms of azole accumulation that are associated with STK-17. As described above, STK-17 coordinately regulates ergosterol biosynthesis and azole accumulation under azole stress.

How STK-17 exerts such a broad effect on the azole resistance is unclear. One explanation is that STK-17 regulates membrane function and homeostasis. A group of membrane genes, including those encoding INSIG domain-containing protein (NCU07869), fatty acid elongase (NCU08976), and neutral ceramidase (NCU04721), were transcriptionally altered by the deletion of *stk-17*, mostly by upregulation of expression, and some of them could be induced by azoles, which impair cell membranes. Thus, it is possible that *stk-17* deletion prevents proper functioning of the cell membrane. In S. cerevisiae, the homolog of STK-17, Vhs1p, physically interacts with diacylglycerol and phosphatidylethanolamine (40), suggesting a potential interaction between STK-17 and the cell membrane. Ergosterol homeostasis is an important aspect of membrane homeostasis. Azole accumulation is also affected by the function and integrity of the cell membrane. Membrane lipid raft disruption was previously shown to be associated with intracellular miconazole accumulation (41). Deletion of the ergosterol biosynthesis gene, *ERG6*, in yeast affects the rate of passive drug diffusion across the membrane, without affecting Pdr5p-mediated drug export (42). In Candida glabrata, reduced activity of Erg1p, another enzyme in ergosterol biosynthesis, increases fluconazole import (43). In spite of these studies, the effects of *stk-17* deletion on cell membrane integrity is largely unknown. It seems to be specifically associated with sterol metabolism, since the deletion only resulted in hypersensitive to ergosterol biosynthesis inhibitors but not to inhibitors of fatty acid and sphingolipid biosynthesis. This may mean that STK-17 functionally interacts with sterol-enriched membrane regions (44), but we did not find any differences between WT and *stk-17*^KO^ strain by using filipin III to stain membrane sterols (unpublished data). Further investigations are needed.

Deletion of *stk-17* had pleiotropic effects, primarily related to azole sensitivity and oxidative stress response. It may be that the hypersensitive to azoles of the *stk-17^KO^* strain is a result of oxidative stress because some azoles do induce oxidative stress (25, 26). However, only fungicidal azoles such as miconazole can induce oxidative stress (26), while most other azoles like ketoconazole and fluconazole used are fungistatic (2). In addition, the genes required for coping with oxidative stress are not necessarily required for azole resistance even the one (*cat-2*) whose expression is induced by ketoconazole. As well as in fission yeast, deletion of *sty1*, the homologous gene of yeast HOG1, resulted in increased sensitivity to oxidative stress but resistance to azoles (45, 46). Moreover, we found that antioxidant N-Acetylcysteine (NAC) could not make N. crassa resistant to azoles, which is different from the results in Aspergillus fumigatus (47). This would be due to the differences between fungi. But we noticed that NAC addition without pH adjustment resulted in azole resistance (our unpublished data). Further experiments involving comparison between different fungi may give a comprehensive explanation. Above all, STK-17’s regulation of resistance to azoles may be independent of its effects on oxidative stress. Although, the sensitivity of *stk-17*^KO^ strain to amphotericin B is possibly associated with oxidative stress. Amphotericin B directly extract ergosterol in the membrane and induces iron leakage and oxidative stress subsequently (48). As ergosterol content was not changed in *stk-17*^KO^ strain under normal condition, its higher sensitivity to amphotericin B may not be ergosterol dependent and more likely to be associated with oxidative stress.

The hypersensitive phenotype of the *stk-17^KO^* strain to oxidative stress may be linked to STK-17-regulated membrane function. We speculate that the connection between membrane homeostasis and the response of *stk-17^KO^* to oxidative stress may be through *erg11* (lanosterol 14α-demethylase) because a deficiency in lanosterol 14α-demethylase in a C. glabrata strain resulted in enhanced oxidative killing (49). A possible explanation for this would be as a result of the ergosterol located the in mitochondrial outer membrane (50). However, we found that overexpression of *erg11* in WT had no impact on oxidative stress resistance, indicating that it is specific to *stk-17* deletion. Because *erg11* is mainly involved in the biosynthesis of ergosterol, a major component of the membrane, it is possible that STK-17-regulated oxidative stress is associated with membrane function.

Unfortunately, proteins directly downstream of kinase STK-17 have not been identified. A limitation for this identification is the low abundance of STK-17 as the protein cannot be detected even in the overexpression strain. Its targets may also be low abundant and not easily detected in large scale identification through proteomics. We examined the relationship between STK-17 and some transcription factors as an alternative. However, no candidate was obtained. It’s interesting that SREBP and AtrR, two regulators for ergosterol biosynthesis in A. fumigatus (15, 17), were not responsible for azole resistance in N. crassa. It’s also reported in F. graminearum that deletion of SREBP did not lead to azole hypersensitivity (51). So, this may be another example for the regulatory evolution of ergosterol biosynthesis as that found in Saccharomycotina (18). Further investigation may show us some interesting observations.

STK-17-mediated stress resistance is functionally conserved among fungi because homologs of STK-17 in different fungi from yeast to filamentous fungi can restore drug sensitive phenotypes of *stk-17*^KO^ strain. However, our results suggest the possibility of functional divergence on the part of STK-17 as well. STK-17 homologs in yeast cannot restore the conidiation phenotype to the *stk-17*^KO^ strain. In addition, deletion of genes encoding the STK-17 homologs in *F. verticilioides* showed no defects in conidiation. In A. nidulans, no defects were found in STK-17 mutants, including conidiation (52). Moreover, several phenotypes of yeast Vhs1p, such as sensitive to hyperosmotic stress, were not evident in N. crassa (53). Thus, the functions of STK-17 in fungi seem to be divergent throughout evolution. Supported by our results of heterologous complementation experiment, this divergence could be a consequence of changes in kinase substrate specificity and alteration of phosphorylation events in fungi (54). Further investigation should provide more information on the modification of regulatory networks in fungi. Still, it is clear that STK-17 and its homologs play important roles in fungal physiology, such as pseudo hyphal growth in C. albicans and yeast (34), glucose starvation in yeast (34), meiosis in fission yeast (35), and conidiation and stress response in N. crassa, thus making it a promising antifungal target.

## MATERIALS AND METHODS

### Strains and culture conditions.

N. crassa strains used in this study are listed in Table S1A. The WT strain FGSC#4200 and the *stk-17* deletion mutant FGSC#18049 (*stk-17^KO^*) were obtained from the Fungal Genetics Stock Center (www.fgsc.net; University of Kansas Medical Center). The double deletion mutants were created by crossing the corresponding single deletion mutants. All the strains were cultured on Vogel’s medium (Vogel’s minimum medium, supplemented with 2% sucrose for slants or glucose for plates, and 1.5% agar) (55). Regeneration medium was used for protoplast transformation and consisted of Vogel’s minimum medium plus 1 M sorbitol, 0.5 g/L fructose, 0.5 g/L glucose, 20 g/L sorbose, and 1.5% agar. Sorbose medium was used for electroporation and consisted of Vogel’s minimum medium with 20 g/L sorbose, 0.5 g/L fructose, 0.5 g/L glucose, and for solid medium, 1.5% agar (56). All cultures were grown at 28°C. Hygromycin B (150 mg/L; Amresco) and chlorimuron ethyl (15 mg/L; Sigma) were added as needed.

YPG liquid medium (0.3% yeast extract, 1% peptone, and 2% glucose) and PDA medium (20% potato, 2% glucose, and 1.5% agar) were used for culturing the F. verticillioides strains listed in Table S1A. Protoplasts of F. verticillioides were recovered on regeneration medium (yeast extract 0.1%, casein hydrolysate 0.1%, sucrose 0.8 M, agar 1.6%) after transformation. All F. verticillioides cultures were grown at 28°C. Hygromycin B at a final concentration of 150 mg/L was added as needed.

### Drug susceptibility test.

N. crassa strains were cultured for 5 to 7 days and conidia were then washed with sterile water to generate conidial suspension (2 × 10^6^ per mL). Two microliter of conidial suspension were inoculated onto the center of Vogel’s plates (diameter Φ = 9 cm) with or without antifungal drugs. All the plates were then incubated at 28°C. Antifungal azoles were dissolved in dimethyl sulfoxide (DMSO) and added to the Vogel’s medium before plating. The antifungals were tested at final concentrations of 2.0 μg/mL for ketoconazole (KTC, Sigma), 0.5 μg/mL for voriconazole (VOR, Sigma), 25 μg/mL for fluconazole (Sigma), and 10 μg/mL for itraconazole (Sigma). For other drugs or stresses, the final concentrations were 5 μg/mL for menadione (Sigma), 0.25 μg/mL for amorolfine (J&K), 2 μg/mL for terbinafine (J&K), 0.025 μg/mL for amphotericin B (Sigma), 10 μg/mL for cerulenin (Enzo Life Sciences), 20 ng/mL for myriocin (Enzo Life Sciences), 8% for NaCl, 1 μg/mL for caspofungin (Apexbio), and 0.005% for SDS (Sangon Biotech). Colonies of the strains were sampled and documented at various time points during growth.

F. verticillioides conidia from 7-day-old cultures were suspended in sterile water and the concentration was adjusted to 10^7^ per mL, and diluted to 10^6^, 10^5^, and 10^4^ conidia per mL. Two microliter of each conidial suspension were inoculated onto PDA plates (diameter Φ = 15 cm) with or without the antifungal drugs, posaconazole (ApexBio), VOR, and itraconazole, all at a concentration of 1 μg/mL. The plates were then incubated at 28°C for 72 h and photographed.

### Plasmid construction and transformation.

Plasmid pCB1532 containing the chlorimuron ethyl resistance gene was used to construct the plasmid pCOMstk-17 for *stk-17^KO^* strain complementation. Briefly, a 4,608-bp fragment containing the *stk-17* coding sequence (1,616 bp) flanked by a 1,430-bp upstream regulatory region and a 1,561-bp downstream region was amplified using primers Pstk-17F and Tstk-17R (Table S1B) and inserted into pCB1532 after being digested by *XbaI* and *Hin*dIII.

The plasmid expressing 5×myc-6×his-tagged STK-17 was also constructed to complement the *stk-17^KO^* strain. The upstream and downstream regions of *stk-17* were first amplified using primer pairs, Pstk-17F/Pstk-17R and Tstk-17F/Tstk-17R, respectively (Table S1B) and inserted into pCB1532 digested with *XbaI* and *Hin*dIII, while the upstream and downstream sequences were double digested with enzyme pairs *XbaI*/*Eco*RI and *Eco*RI/*Hin*dIII, respectively. This resulted in the plasmid pCB1532-up-down. The 5×myc-6×his was tagged to the N-terminus of STK-17 by simply inserting the coding sequence of *stk-17* into the Qa plasmid (57) using primers, stk-17F and stk-17R (Table S1B). The tagged sequence was then amplified using primers, Tagstk-17F and Tagstk-17R (Table S1B), digested with *Eco*RI and inserted into EcoRI digested pCB1532-up-down, resulting in the complementary plasmid pTCOMstk-17.

The kinase-dead (KD) plasmid was constructed from pTCOMstk-17. The primers stk-17KD-F and stk-17KD-R (Table S1B) containing the KD point mutation (D183A) were designed, and the first half and second half of 5×myc-6×his tagged STK-17 flanked with the native promoter and terminator of *stk-17* were respectively amplified with the primer pairs, Pstk-17F/stk-17KD-R and stk-17KD-F/Tstk-17R (Table S1B) from pTCOMstk-17. The two sequences were then fused by overlap-extension PCR because primers stk-17KD-F and stk-17KD-R were complementary. The final plasmid pKDstk-17 was constructed by inserting an *XbaI*- and *Hin*dIII-digested fusion fragment into pCB1532.

An *stk-17* overexpression plasmid was constructed by a method similar to what had been reported previously using the *cfp* promoter (5, 16, 58). The expression of *cfp* gene is not changed by azoles and oxidative stress (6, 59), making its promoter suitable for gene overexpression in our study. Briefly, the minimal *cfp* promoter (58) and *trpC* terminator were amplified using primer pairs PcfpF/PcfpR and TtrpC-F/TtrpC-R, respectively (Table S1B), and inserted into *XbaI*/*Hin*dIII-digested pCB1532, while the upstream and downstream fragments were digested by *XbaI*/*Eco*RI and *Eco*RI/*Hin*dIII, respectively, resulting in the plasmid, pOE-UD. The 5×myc-6×his tagged *stk-17* was then amplified using primers Tagstk-17F and Tagstk-17R (Table S1B), and digested by *Eco*RI and inserted into the *Eco*RI digested pOE-UD, resulting in the plasmid pOEstk-17.

The *erg11* overexpression plasmid was generated from pOEstk-17 by replacing *stk-17* with *erg11*. Briefly, the coding sequence of *erg11* and a new *trpC* terminator were first amplified with primer pairs erg11F/erg11R and TtrpCF’/TtrpCR, respectively, and then inserted into *Asc*I/*Hin*dIII-digested pOEstk-17 after digesting with *Asc*I/*XmaI* and *XmaI*/*Hin*dIII, respectively, resulting in plasmid pOEerg11.

The pCOMstk-17, pTCOMstk-17, pKDstk-17, and pOEerg11 were then transformed into the *stk-17^KO^* strain and/or the WT strain using protoplast transformation or electroporation transformation as previously described (5). The chlorimuron ethyl-resistance gene was used as the selective marker. The transformants were verified by PCR and the primers are listed in Table S1B.

### RNA extraction and gene expression analysis using qRT-PCR.

Samples for RNA extraction were prepared as previously described (32). Briefly, 15 small pieces of mycelial mats (1 to 2 mm^2^) were grown in liquid Vogel’s medium for 13 h and then treated with or without ketoconazole for 12 h or 24 h. Harvested samples were immediately frozen, and then ground into fine powder in liquid nitrogen. Total RNA extraction was performed according to the standard TRIzol protocol (Invitrogen, Carlsbad, CA, USA). The cDNAs were then synthesized from 2 to 3 μg total RNA using the FastQuant RT kit with gDNase (Tiangen, China) according to the manufacturer's protocol, and diluted to a final volume of 50 μL.

Gene-specific primers for qRT-PCR analysis were designed using online tools PrimerQuest or Primer 6 and listed in Table S1C. The qRT-PCR was performed on a CFX96 multicolor real-time PCR detection system (Bio-Rad, Hercules, CA) with SYBR green detection (SYBR green Realtime PCR Master Mix; TOYOBO, Tokyo, Japan) according to the manufacturer's instructions. At least three independent experiments were carried out and each cDNA sample was analyzed in duplicate. The average threshold cycle (CT) values were used to calculate relative expression levels normalized to expression of β-tubulin using the 2^−ΔΔCT^ method (60).

### RNA-seq and data analysis.

The transcriptomic profiles of the WT strain, the *stk-17^KO^* strain, and the *stk-17^com^* strain were analyzed by RNA-sequencing (RNA-seq) and compared. The mycelia of those strains treated with or without a sub-inhibitory dosage (1 μg/mL) of KTC for 12 h were harvested and RNA was extracted as described above. The total RNA samples were then sent to the Beijing Genomics Institute (Wuhan, China) for RNA quality control, sequencing library construction, single-end RNA-sequencing (BGIseq-500RS platform), and data processing as described before (61). Three biological replicates for each sample were analyzed. The transcript level of a gene was determined by calculating the fragments per kilobase of transcript per million mapped reads (FPKM) and represented as average FPKM from three biological replicates for each sample. Genes with FPKM value <5 in all samples were regarded as low-abundance genes and excluded from subsequent analysis. Genes with log_2_|fold change| levels ≥ 0.67 and FDR (false discovery rate) values <0.001 in comparisons between two samples were considered to be differentially expressed. Because *stk-17^com^* was supposed to be identical to WT, the genes concomitantly affected in WT and *stk-17^com^* versus *stk-17^KO^* were included in the total of STK-17 dependent expression. Venn diagramming and heatmap construction were performed with TBtools (62). GO, KEGG, and functional categories enrichment analysis were performed on DAVID (63).

### HPLC-MS analysis of sterol contents and intercellular ketoconazole.

The samples for sterol extraction were prepared by the same method as for RNA extraction. In order to obtain more stable results of sterol profiles, samples with 24-h KTC treatment were used for the subsequent analysis. The collected mycelia of different strains were heat dried at 80°C and then ground into fine powder for sterol extraction and HPLC-MS analysis as reported previously (29). Ergosterol (J&K Scientific Ltd., China) and KTC standards were run for comparison.

### Detection of ERG11 expression by Western blotting.

The samples for protein extraction were prepared using the same method as for RNA extraction. The collected mycelia were immediately frozen and ground into powder for protein extraction. Proteins were extracted as previously reported (57) and quantified with the 2-D Quant Kit (GE, USA). Western blotting was performed with Wes, a simple western machine (ProteinSimple, USA), according to the manufacturer’s instructions. ERG11 protein was detected using an anti-ERG11 antibody generated by our laboratory. This antibody was tested for specificity and sensitivity and proven before use. β-tubulin antibody was used as a loading control. Standard Western blotting was also used to detect 5×myc-6×his tagged ERG11 with an anti-myc antibody (Abbkine, USA).

### Complementation of N. crassa *stk-17^KO^* strain with STK-17 homologs from S. cerevisiae, A. fumigatus, and *F. verticilioides*.

In order to complement the N. crassa *stk-17^KO^* strain, the STK-17 coding sequence in the plasmid pTCOMstk-17 was replaced with coding sequences of A. fumigatus Ran1 (Afu3g10530), *F. verticilioides* Ran1 (FVEG10227), S. cerevisiae Vhs1p, and S. cerevisiae Sks1p to generate the complementary plasmids. For A. fumigatus *ran1*, the coding region of *ran1* and terminator of *stk-17* were amplified with Afran1comF/R and Tstk-17F’/R, respectively (Table S1B). The amplicons were digested with *Asc*I/KpnI and KpnI/EcoRI, respectively, and inserted into *Asc*I/EcoRI-digested pTCOMstk-17, resulting in pTCOMafran1. For *F. verticilioides ran1*, the coding region of *ran1* and the terminator of *stk-17* were first amplified by PCR using primer pairs Fvran1comF/R and Tstk-17F’’/R’, respectively (Table S1B). Then the obtained fragments were inserted into *Asc*I/*Hin*dIII-digested pTCOMstk-17 by homologous recombination using the ClonExpress MultiS one-step cloning kit (Vazyme, Nanjing, China), resulting in pTCOMfvran1. For S. cerevisiae *VHS1*, the coding region of *VHS1* and the terminator of *stk-17* were first amplified by PCR using primer pairs ScVHS1comF/R and Tstk-17F’’/R’, respectively (Table S1B). Then the amplicons were inserted into *Asc*I/*Hin*dIII-digested pTCOMstk-17 by homologous recombination, resulting in pTCOMScVHS1. For S. cerevisiae *SKS1*, the coding region of *SKS1* and the terminator of *stk-17* were first amplified by PCR using primer pairs ScSKS1comF/R and Tstk-17F’’/R’, respectively (Table S1B). Then the amplicons were inserted into *Asc*I/*Hin*dIII-digested pTCOMstk-17 by homologous recombination, resulting in pTCOMScVHS1. These plasmids were then transformed into N. crassa *stk-17^KO^* by electroporation, and the chlorimuron ethyl resistance gene was used as the selective marker. The transformants were verified by PCR.

### Gene deletion in F. verticillioides.

Target gene deletion of F. verticillioides was performed using the homologous recombination method, and the hygromycin B resistance gene *hph* was used as a selective marker. Briefly, hygromycin B resistance gene *hph* was flanked by the 1,433-bp upstream regulatory region and the 1,457-bp downstream region of *Fvran1*, the homologous gene of N. crassa *stk-17*, using overlap-extension PCR. The fused fragment was purified and transformed into protoplasts of F. verticillioides WT strain FGSC#7600 as previously described (6). Transformants were verified by PCR using the primers listed in Table S1B.

### Data availability.

RNA-seq data supporting the results of this article are included in the supplemental material. The raw sequencing data for RNA-seq have been submitted to the Gene Expression Omnibus (GEO) with accession number GSE193984.
