# Supplementary material for: Coordinated Regulation of Membrane Homeostasis and Drug Accumulation by Novel Kinase STK-17 in Response to Antifungal Azole Treatment
Source: Microbiol Spectr. 2022 Feb 23;10(1):e00127-22. doi: 10.1128/spectrum.00127-22 (PMC8865411; doi:10.1128/spectrum.00127-22)
Supplement: SUPPLEMENTAL FILE 1 — Supplemental material. Download SPECTRUM00127-22_Supp_1_seq3.pdf, PDF file, 0.2 MB [file spectrum00127-22_supp_1_seq3.pdf]

Suppl Table 1 Strains and primers used in this study

A Strains

Species	Strain	Genotype	Source
N. crassa	WT	74-ORs-6a	FGSC
	ccg-8^{KO}	Δ*ccg-8*	FGSC
	cdr-4^{KO}	Δ*cdr4*	FGSC
	sah-2^{KO}	Δ*sah-2*	FGSC
	rbd-2^{KO}	Δ*rbd-2*	FGSC
	stk-17^{KO}	Δ*stk-17*	FGSC
	stk-17^{com}	Δ*stk-17*; *stk-17*	This study
	stk-17^{Tcom}	Δ*stk-17*; 5×myc-6×his-*stk-17*	This study
	stk-17^{D183A}	Δ*stk-17*; 5×myc-6×his-*stk-17*^{D183A}	This study
	Af*ran1*^{com}	Δ*stk-17*; 5×myc-6×his-Af*ran1*	This study
	Fv*ran1*^{com}	Δ*stk-17*; 5×myc-6×his-Fv*ran1*	This study
	Sc*VHS1*^{com}	Δ*stk-17*; 5×myc-6×his-Sc*VHS1*	This study
	Sc*SKS1*^{com}	Δ*stk-17*; 5×myc-6×his-Sc*SKS1*	This study
	stk-17^{KO}; *cdr4*^{KO}	Δ*stk-17*; Δ*cdr4*	This study
	stk-17^{KO}; *ccg-8*^{KO}	Δ*stk-17*; Δ*ccg-8*	This study
	stk-17^{KO}; *erg-11*^{oe}	Δ*stk-17*; P*cfp*::*erg11*	This study
	erg-11^{oe}	P*cfp*::*erg11*	This study
F. verticillioides	WT	Wild type	FGSC
	ran1^{KO}	Δ*ran1*	This study

B Primers used for plasmid construction

**Primer**	**Sequences (5'→3')**	**Restriction enzyme**	**Purpose**
Pstk-17F	GCTCTAGAGGCAAGATGGAGAGGCAGTGAC	*Xba*I	
Pstk-17R	GGAATTCGATCTGTCCTTGCTCCTTGACTCC	*Eco*RI	
Tstk-17F	GGAATTCCGGTCGCAAGATTTCGCAATACG	*Eco*RI	
Tstk17F'	GGGGTACCCGGTCGCAAGATTTCGCAATACG	*Kpn*I	
Tstk-17F"	GAATTCCGGTCGCAAGATTTCGCAATACG	NA	
Tstk-17R	CCCCAAGCTTGTGACAGTGGGTGGTGTGGGTA	*Hin*dIII	
Tstk-17R'	tcgacggtatcgataagcttGTGACAGTGGGTGGTGTGGGT	NA	
stk-17F	GGCGCGCCTATGCATCATGTGTCAGAGCTTGGA	*Asc*I	
stk-17R	GGAATTCCTAATAGCAGCCTTGGATATGCGC	*Eco*RI	
Tagstk-17F	GGAATTCACAgGATCTGATATCATCGATTTAAAGCAATG	*Eco*RI	
Tagstk-17R	GGGAATTCCTAATAGCAGCCTTGGATATGC	*Eco*RI	
stk-17KD-F	GAATTTACCACCGGGACTTGAAGC	NA	
stk-17KD-R	GCTTCAAGTCCCGGTGGTAAATTC	NA	
Afran1comF	TTGGCGCGCCAATGCTCACCCCAAATCCCTTCG	*Asc*I	*stk-17* mutant complementation and point mutation
Afran1comR	GGGGTACCCGACATGCCTTAGCAGACGCATAG	*Kpn*I	
Fvran1com1F	caccaccatcatcatggcgcgccAATGCAGCAACATGCCATATTCGG	NA	
Fvran1com1R	AGCAGAGTGGTCCAGGCATTCTGGACTCATGTAGAATG TTGAACCG	NA	
Fvran1com2F	AATGCCTGGACCACTCTGCTAG	NA	
Fvran1com2R	AAATCTTGCGACCGGAATTCGACCTTAGTAACAACCTT GGAACGTAG	NA	
ScVHS1comF	caccaccatcatcatggcgcgccAATGATGATGTTCCATAATTGCAG AATTAATAACTAC	NA	
ScVHS1comR	AAATCTTGCGACCGGAATTCTTAGTTAGTTAAAGAAGA GGAACTCATGCG	NA	
ScSKS1comF	caccaccatcatcatggcgcgccAATGCTGTCAGACTGCTTGCTGA AC	NA	
ScSKS1comR	AAATCTTGCGACCGGAATTCCTGCTCAATAATCTGGTAA CCATTTCTCG	NA	
stk-17vF	GCTATGCCGTCAAGTGCCTCAG	NA	
stk-17vR	GGAGCGTGGTGTGAATGCGTAT	NA	
cdr4vF	TCCTGCTCGTGTCCTTCGTCACTG	NA	Knock out or double mutant verify
cdr4vR	TCAATGCAATGCATGACCATCAACTTC	NA	
ccg-8vF	TCAGAAGCTCCGACGCTTGGACAC	NA	
ccg-8vR	TCAAAGGTCGAATCTCCTGCTCTGTG	NA	
PcfpF	GCTCTAGAGCTCAGCCCACGTTGCCATT	*Xba*I	
PcfpR	GGAATTCGGGTGAGATCTGGTGGTGAAGAAG	*Eco*RI	
TtrpC-F	GGAATTCCCGGGATCCACTTAACGTTACTGAA	*Eco*RI	*erg-11* overexpression
TtrpC-F'	TCCCCCCGGGATCCACTTAACGTTACTGAA	*Xma*I	
TtrpC-R	CCCCAAGCTTGGCGTAGAGGATCCTCTAGAAAGAAG	*Hin*dIII	
erg-11F	TTGGCGCGCCTATGGGCATCCTGCAAGTCGTTG	*Asc*I	

erg-11R	TCCCCCCGGGCCTCACCACCACATCAGTGTCTTTAC	*Xma*I	
Fvran1f5-F	GAGTGAGTACCGAGTACGCCTAC	NA	
Fvran1f5-R	CATCTTCTGTCGTGGGTTGTATTCTCTGGTTTGAGG	NA	
Fvran1f3-F	TGCCGACCGGATCTCTCGCCTTCGACATAATGG	NA	
Fvran1f3-R	GTCTCCTCCTCATCACGCTACC	NA	
hph-F	GAATACAACCCACGACAGAAGATGATATTGAAGGAGC	NA	
hph-R	CGAGAGATCCGGTCGGCATCTACTCTATTCCT	NA	Fv*ran1* deletion and verify
Fvran1v1	GCAACAGTGGTGCCAGTTCAC	NA	
Fvran1v2	GCCGCCGCTACTGCTTACAA	NA	
Fvran1v3	TGGCTTGTATGGAGCAGCAGAC	NA	
Fvran1v4	CACGTTGAGTGGTCGGCAGTT	NA	
Fvran1v5	GCAGCAACATGCCATATTCGGT	NA	
Fvran1v6	GGAGCTTGCACAGAGACAGGAA	NA	

C Gene specific Primers used for qRT-PCR

Gene	Forward primer(5'→ 3')	Reverse primer(5'→ 3')
β-tubulin	CCCAAGAACATGATGGCTGCTTCT	TTGTTCTGAACGTTGCGCATCTGG
NCU04156	TGAGCACCTTCACGATCTGTCCAA	TGATGTACATAGCACCCATGGCAC
NCU05278	TTTCACCTTCCTCTTCGCTTCCCA	TCATCGACTCAAGCTGCTCCATGT
NCU02624	AAATCGATTACGGCTACGGTCTCG	TATCGCTACCATCCACGTTCCTGA
NCU05591	GCTTTGGAAATGGATGGTGACGCT	AAATGCAGAGGGCGGTCTTAGAGT
NCU03006	TCAGCTCAAGTTCGTCAAGGGTGA	TTCATAGACACCAAAGGTACCGCC
NCU08762	TCATCGCCAAGCAATACC	CTCCGAAACTCAGCATGAA
