# Supplementary material for: Coordinated Regulation of Membrane Homeostasis and Drug Accumulation by Novel Kinase STK-17 in Response to Antifungal Azole Treatment
Source: Microbiol Spectr. 2022 Feb 23;10(1):e00127-22. doi: 10.1128/spectrum.00127-22 (PMC8865411; doi:10.1128/spectrum.00127-22)
Supplement: SUPPLEMENTAL FILE 3 — Supplemental material. Download SPECTRUM00127-22_Supp_3_seq2.pdf, PDF file, 3.1 MB [file spectrum00127-22_supp_3_seq2.pdf]

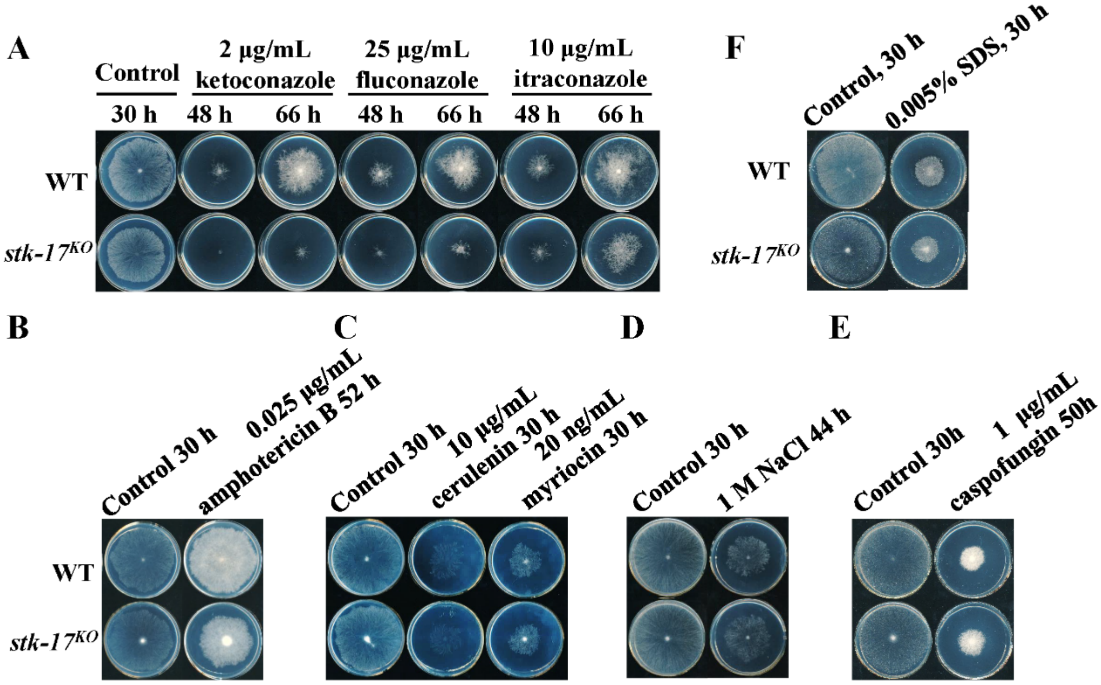

Suppl Figure 1. STK-17 is involved in the resistance to ergosterol biosynthesis inhibitors. (A) Sensitivity of wild type (WT) and *stk-17*^{KO} strain to ketoconazole, fluconazole and itraconazole. (B) Sensitivity of WT and *stk-17*^{KO} strain to ergosterol binding agent amphotericin B. (C) Sensitivity of WT and *stk-17*^{KO} strain to cerulenin and myriocin. (D) Sensitivity of WT and *stk-17*^{KO} strain to osmotic stress induced by NaCl. (E) Sensitivity of WT and *stk-17*^{KO} strain to cell wall biosynthesis inhibitor caspofungin. (F) Sensitivity of WT and *stk-17*^{KO} strain to detergent SDS. Two microliter conidia suspensions (2×10^6 conidia/mL) for each strain were inoculated on Vogel's plates with or without indicated chemicals. The plates were then incubated at 28°C and the colonies were documented at indicated time point.

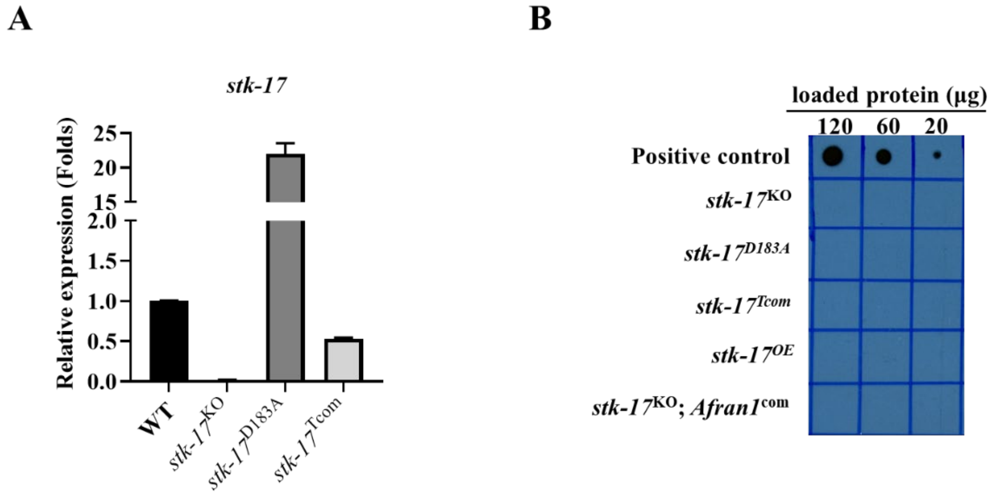

Suppl Figure 2. Transcript and protein levels of STK-17 various strains. (A) Transcript levels of *stk-17* in WT, *stk-17*^{KO}, *stk-17*$^{\mathrm{D183A}}$ and *stk-17*^{Tcom} strain. After grown in liquid Vogel's medium for 24 h, the mycelium of indicated strains were harvested for total RNA extraction. The transcript levels of *stk-17* were measured by quantitative real-time polymerase chain reaction (qRT-PCR), and the expression was calculated by $2^{-\Delta\Delta Ct}$ method and normalized to *β-tubulin*. (B) Dot assay of *myc* tagged STK-17 in *stk-17*^{KO}, *stk-17*$^{\mathrm{D183A}}$, *stk-17*^{Tcom}, *stk-17*^{OE}, and *stk-17*^{KO}; *Afran1*^{com} strain using an anti-myc antiserum. Protein of myc-cot was used as a positive control.

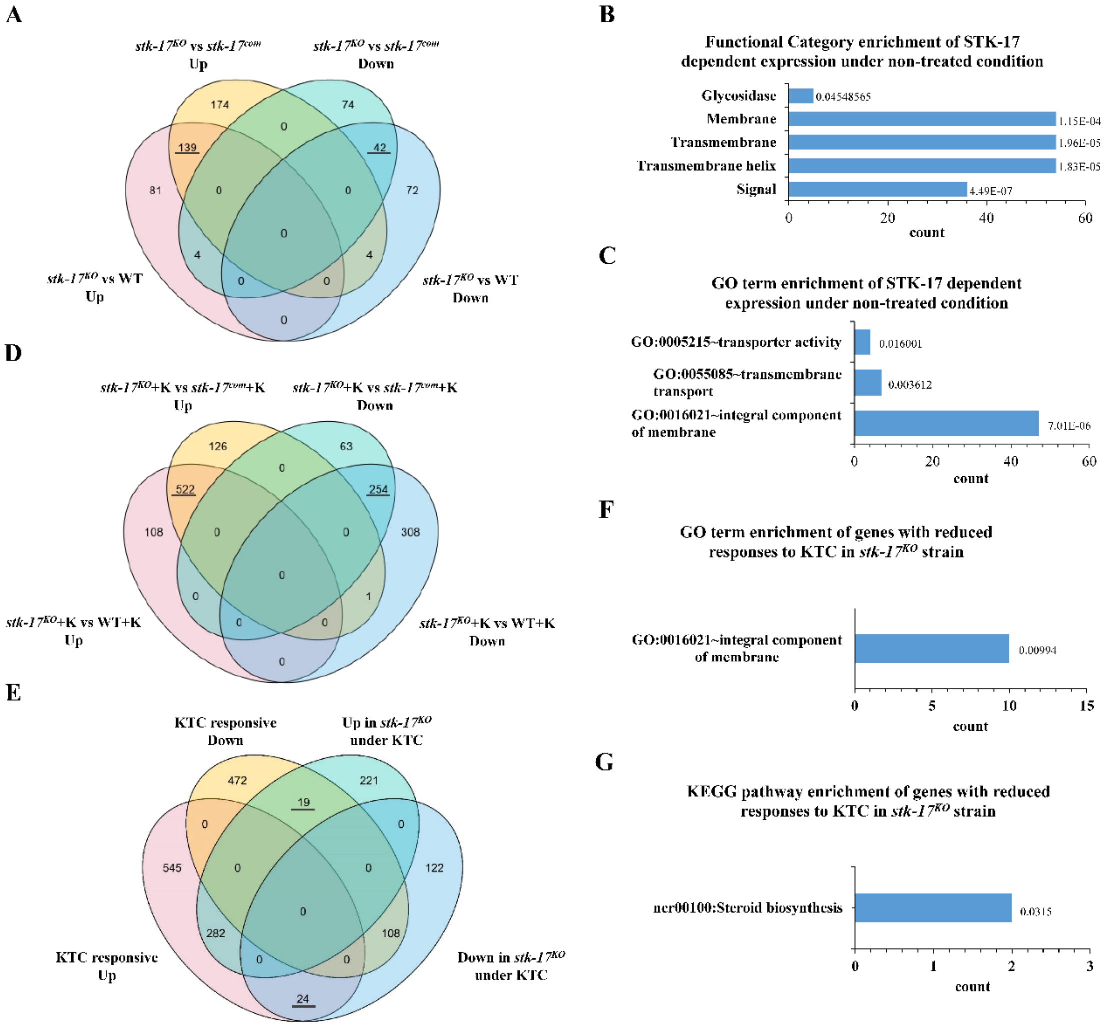

Suppl Figure 3. Deletion of *stk-17* affects expression of membrane proteins. (A) Identification of STK-17 dependent genes under non-treated condition. Genes concomitantly affected by *stk-17* deletion when compared to wild type (WT) and *stk-17*^{COM} strain were identified as STK-17 dependent genes, which were indicated by underlines. (B) and (C) DAVID Functional Category and GO term enrichment analysis of STK-17 dependent genes under non-treated condition. Number beside each bar indicates the q-value for the enriched GO terms. (D) Identification of STK-17 dependent genes under ketoconazole (KTC)-treated condition. (E). Veen analysis of KTC-responsive genes and STK-17 dependent genes under KTC-treated condition. Number underlined indicates numbers of gene with reduced responses to KTC. GO term enrichment analysis (F) and KEGG enrichment analysis (G) were performed for these genes with reduced responses to KTC.

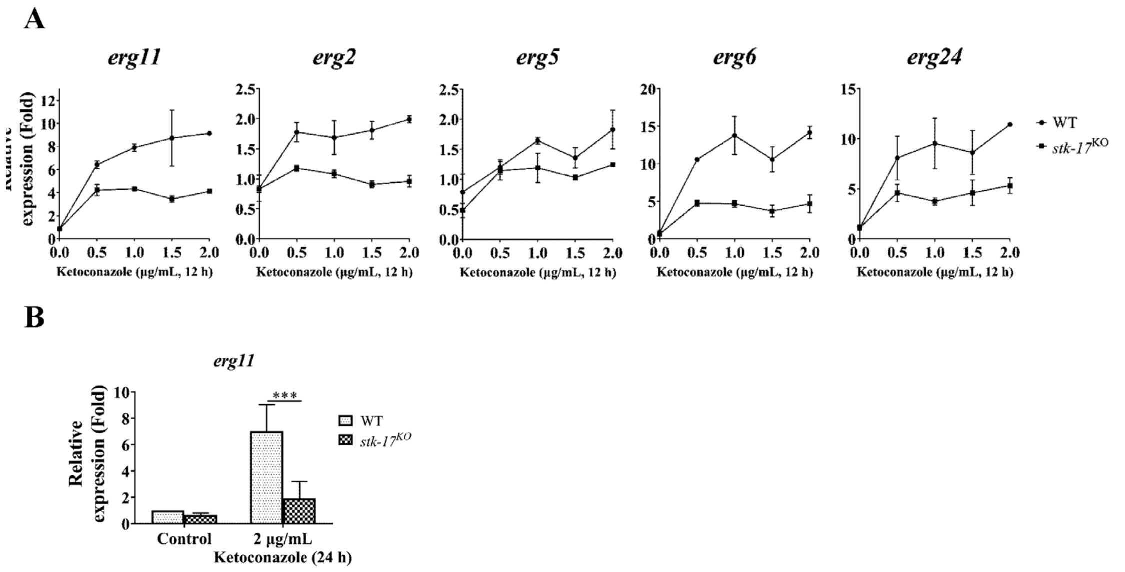

Suppl Figure 4. STK-17 is required for the responses of *erg* genes to ketoconazole. (A) After grown in liquid Vogel's medium for 13.5 h, the mycelium of wild type (WT) and *stk-17*^{KO} strain were treated with various concentrations of Ketoconazole for additional 12 h. The transcript levels of *erg11* and *erg2*, *erg5*, *erg6*, *erg24* were measured by quantitative real-time polymerase chain reaction (qRT-PCR), and the expression was calculated by $2^{-\Delta\Delta Ct}$ method and normalized to *β-tubulin*. (B) After grown in liquid Vogel's medium for 13.5 h, the mycelium of WT and *stk-17*^{KO} strain were treated with 2 μg/mL Ketoconazole for additional 24 h. The transcript levels of *erg11* were measured by qRT-PCR, and the expression was calculated by $2^{-\Delta\Delta Ct}$ method and normalized to *β-tubulin*. The results presented here are means of three biological replicates, and the significant levels were calculated by t-test and marked as *($p<0.05$), **($p<0.01$) or ***($p<0.001$).

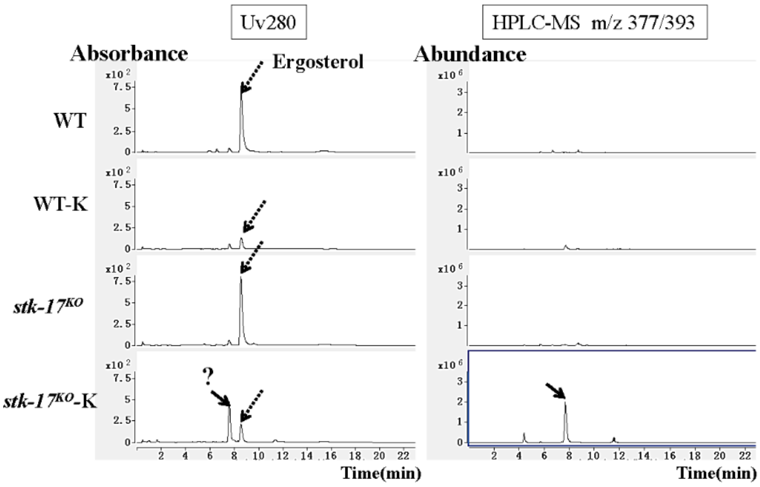

Suppl Figure 5. Deletion of *stk-17* leads to the accumulation of an unknown sterol. After grown in liquid Vogel's medium for 13.5 h, the mycelium of wild type (WT) and *stk-17*^{KO} strain were treated with ketoconazole (K). The total sterols were extracted and analyzed by HPLC-MS. The left panel shows the detection of sterols through Uv280 absorption. Dotted arrows indicate the peak of ergosterol and arrow marked with question mark indicates the unknown sterol. The right panel shows the MS detection of the unknown sterol. This sterol has a m/z of 377 or 393 and indicated by an arrow.

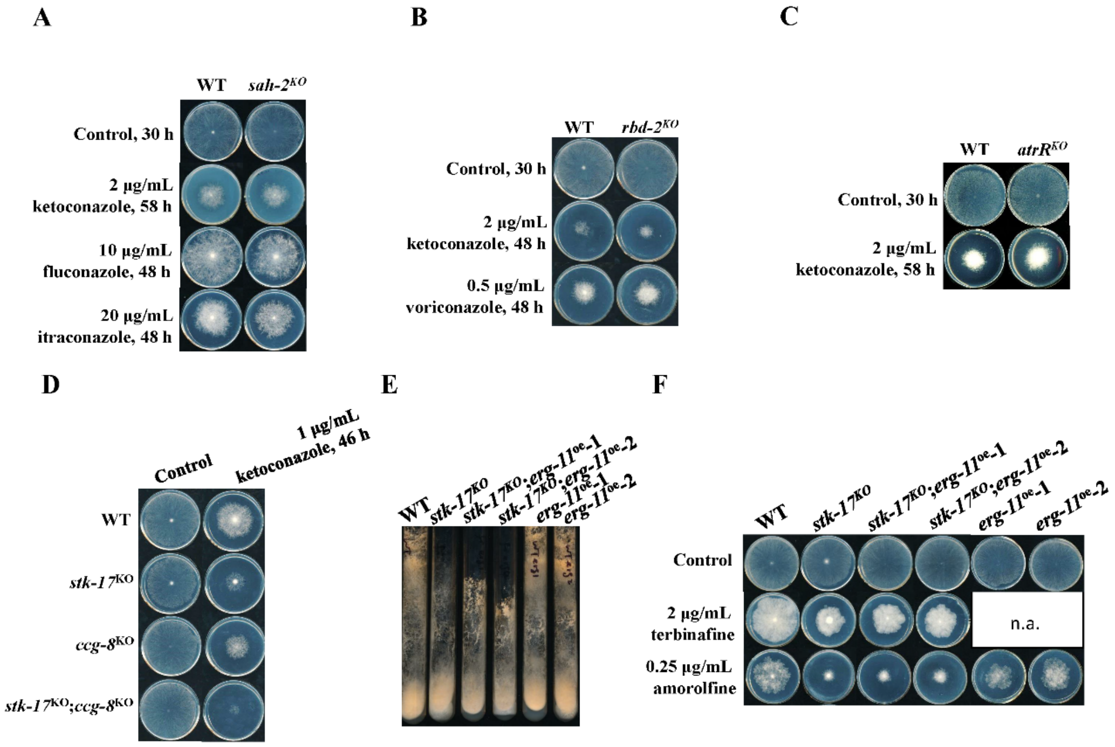

Suppl Figure 6. STK-17 has a genetic interaction with ERG11. (A) and (B) Sensitivity test of WT, *sah-2*^{KO} and *rbd-2*^{KO} strain to various azoles. Two microliter conidia suspensions (2×10^{6} conidia/mL) for each strain were inoculated on Vogel's plates with or without indicated chemicals. The plates were then incubated at 28°C and the colonies were documented at indicated time point. (C) Sensitivity test of WT, *atrR*^{KO}strain to ketoconazole. (D) Sensitivity test of WT, *stk-17*^{KO}, *ccg-8*^{KO} strain and *stk-17*^{KO}; *ccg-8*^{KO} strain to ketoconazole. (E) Phenotypes of WT, *stk-17*^{KO} strain and *erg11* overexpression strains grown on Vogel's slants. (F) Sensitivity of WT, *stk-17*^{KO} strain and *erg11* overexpression strains to terbinafine and amorolfine. Two microliter conidia suspensions (2×10^{6} conidia/mL) for each strain were inoculated on Vogel's plates with or without indicated chemicals. The plates were then incubated at 28°C and the colonies were documented at indicated time point.

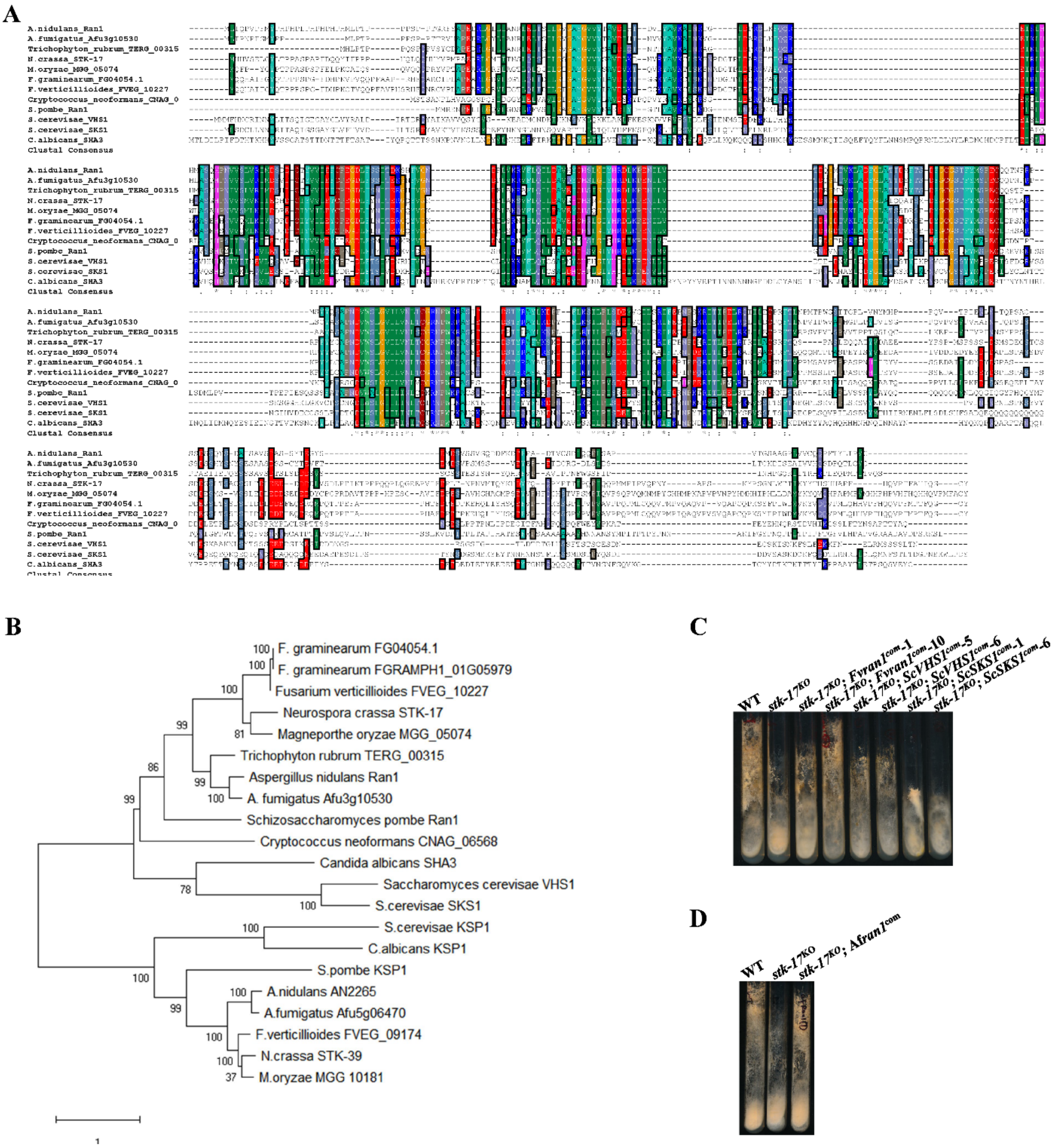

Suppl Figure 7. STK-17 are widely distributed and functionally conserved among fungi. (A) Amino acid sequences of STK-17 homologs were retrieved from NCBI through blast analysis. Fungal species analyzed includes *Aspergillus fumigatus*, *Aspergillus nidulans*, *Trichophyton rubrum*, *Magnaporthe oryzae*, *Fusarium graminearum*, *Fusarium verticilioides*, *Cryptococcus neoformans*, *Schizosaccharomyces pombe*, *Saccharomyces cerevisiae* and *Candida albicans*. Alignment was performed with ClusterX2 and visualized by BioEdit. (B) The phylogenic tree of STK-17 and its homologs were conducted in MEGA X by using the Maximum Likelihood method with JTT+G+I model. The tree with the highest log likelihood is shown. The percentage of trees in which the associated taxa clustered together is shown next to the branches. Since STK-39 and STK-17 belongs to the same kinase subfamily, STK-39 clade was used as outgroup. (C) and (D) The phenotypes of *stk-17*^{KO} strain and its complemented strains grown on Vogel's slants. The complemented strains includes *Fvran1* complemented *stk-17*^{KO} strains, *Afran1* complemented *stk-*

17^{KO} strains, *ScVHS1* phylogenic complemented *stk-17*^{KO} strains and *ScSKS1* complemented *stk-17*^{KO} strains. The conidia of each strain were inoculated on Vogel's slants. After grown for 7 days, the production of conidia were documented.
